# Personal care products disrupt the human oxidation field

**DOI:** 10.1126/sciadv.ads7908

**Published:** 2025-05-21

**Authors:** Nora Zannoni, Pascale S. J. Lakey, Youngbo Won, Manabu Shiraiwa, Donghyun Rim, Charles J. Weschler, Nijing Wang, Tatjana Arnoldi-Meadows, Lisa Ernle, Anywhere Tsokankunku, Gabriel Bekö, Pawel Wargocki, Jonathan Williams

**Affiliations:** ^1^ Atmospheric Chemistry Department, Max Planck Institute for Chemistry, Mainz, Germany.; ^2^ Department of Chemistry, University of California, Irvine, CA, USA.; ^3^ Department of Architectural Engineering, Pennsylvania State University, University Park, PA, USA.; ^4^ International Centre for Indoor Environment and Energy, Department of Environmental and Resource Engineering, Technical University of Denmark, Lyngby, Denmark.; ^5^ Environmental and Occupational Health Sciences Institute, Rutgers University, Piscataway, NJ, USA.; ^6^ Energy, Environment and Water Research Center, The Cyprus Institute, 1645 Nicosia, Cyprus.

## Abstract

People generate hydroxyl radicals (OH) in the presence of ozone via the ozonolysis of skin-emitted alkenes. In this study, we found that the application of personal care products (PCPs) including fragrances and body lotions suppresses the human oxidation field. Body lotion hampers the generation of 6-methyl-5-hepten-2-one, a key OH precursor, while many volatile ingredients of PCPs enhance OH loss in the gas phase. Although fragrances contain terpenes capable of generating OH through ozonolysis, the much larger amount of ethanol solvent acts as a large OH sink. We combined a multiphase chemical kinetic model and a computational fluid dynamics model to demonstrate how the concentrations of the reactive components develop in the indoor environment. These findings have implications for the indoor chemistry of occupied spaces and human health.

## Teaser

Personal care products substantially suppress a human’s production of OH radicals.

## INTRODUCTION

The occupied indoor environment contains multiple sources of chemical compounds (*1*). These include not only continuous emissions from housing materials such as furniture, floors, and furnishings but also periodic intense emissions from human activities such as cooking, smoking, and cleaning. Outdoor air chemicals can also enter indoor environments through passive and active ventilation. Ozone (O_3_) from outdoors can react with compounds indoors to create a complex chemical cocktail within the indoor living space (*1*). Because people spend up to 90% of their time indoors (*2*), exposure to this diverse array of chemical compounds over extended periods is cause for concern, particularly as the human health impacts of many such chemicals remain poorly understood (*3*).

Recent work has revealed that human beings themselves are potent sources and sinks of chemical compounds indoors (*4*–*17*). Zannoni *et al.* (*11*) have shown that O_3_ reactions on skin, and subsequently with products in the air, create a body-enveloping oxidation field of OH radicals. At ~35 parts per billion (ppb) of O_3_ in a 22.5-m^3^ room ventilated at ~3 air changes per hour, the OH concentration generated by one young adult is ~2 × 10^5^ molecules cm^−3^ (*11*), comparable to outdoor levels in some locations (*18*). Hydroxyl radicals are extremely reactive, capable of oxidizing a wider range of species than O_3_, typically at faster rates (*19*–*21*). Such a natural oxidation field can alter personal chemical exposures indoors.

The discovery of the personal oxidation field was made by exposing volunteers to O_3_ under tightly controlled conditions with restricted use of personal care products (PCPs). However, real-world scenarios include cleaning agents and scented products whose terpene constituents can also react with O_3_ to generate OH radicals (*22*–*26*). Other chemicals emitted indoors (e.g., alkanes from cooking oils) react with OH radicals, reducing their concentration (*27*). The most direct potential influence on the OH field intensity comes from PCPs such as skin lotion and fragrances. Globally, PCP usage is widespread, with a current estimated annual revenue of US $646.2 billion in 2024 (*28*). Such extensive consumption has been shown to even affect outdoor air quality in densely populated regions in North America and Europe (*29*, *30*).

Given that the human oxidation field influences the chemical composition of air in the breathing zone and close to the skin, it affects our intake of chemicals, which, in turn, affects human health. It is therefore of interest to examine how PCPs can influence the strength and spatial extent of the self-generated OH field. Within the framework of the ICHEAR (Indoor Chemical Human Emissions and Reactivity) project (*5*), we evaluated the influence of two different PCPs on the human oxidation field. Four young adults were first exposed to O_3_ in a temperature-controlled indoor environment, and then the experiment was repeated after the application of a widely used body lotion or fragrance (fig. S1). By combining air measurements from within the chamber with model simulations, we determined the net effect of the lotion and fragrance on the human oxidation field.

## RESULTS

### Indoor air chemistry of people wearing a body lotion

**Fig. 1.**
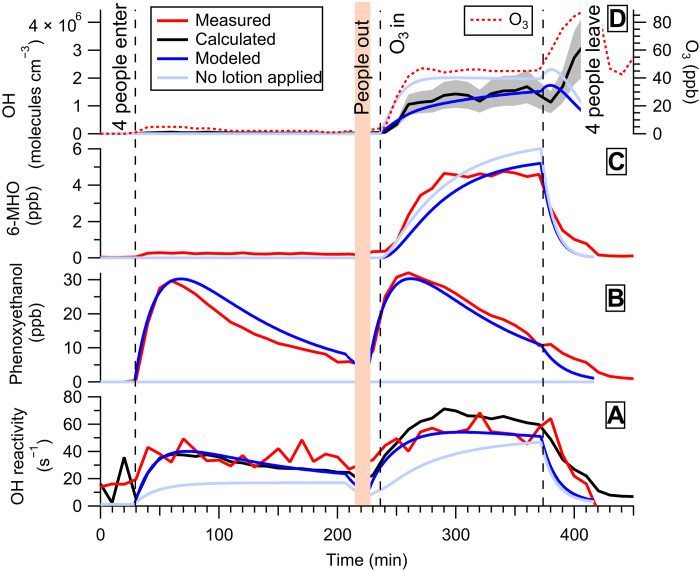
Lotion case study. Four young adult volunteers wearing a body lotion occupy the chamber. Ozone (40 ppb) is mixed with chamber air starting at 240 min. (**A**) OH reactivity. (**B**) Phenoxyethanol concentration. (**C**) 6-MHO concentration. (**D**) OH and O_3_ concentrations. All measured variables are reported in red, calculated variables are in black, and modeled results are in blue. Equations 2 and 3 for calculating the OH reactivity and OH concentrations, respectively, are shown in Materials and Methods (Supplementary Materials). The OH concentration was estimated with the steady-state method. The cyan lines represent the simulated conditions of no lotion being applied during the experiment. The estimated uncertainties on the measurements are 48% (OH reactivity), 10 to 50% (phenoxyethanol and 6-MHO), 35% (calculated OH concentration; shaded gray area), and 2% (O_3_).

We first examine how the application of body lotion affects the chemistry in the periphery of the persons tested. Figure 1 shows OH concentration (in molecules per cubic centimeter), OH reactivity (per second), and selected gas-phase species, including a lotion ingredient phenoxyethanol (in parts per billion) and a skin oxidation product 6-methyl-5-hepten-2-one (6-MHO; in parts per billion). The data were generated on the basis of measurements made while four people wearing a fragrance-free body lotion containing linoleic acid (Neutral, Unilever body lotion for sensitive skin; 0% colorants and 0% perfume) sat inside a chamber. Before entering the chamber (within 5 to 7 min), the body lotion (on average, 5.9 g of lotion on a surface area of 0.516 m^2^) was uniformly applied to the participants’ exposed skin surfaces. The experiments were conducted without O_3_ in the morning and with O_3_ (45 ppb) in the afternoon. Measured, calculated, and modeled OH reactivity agreed within the method uncertainties for both the O_3_-free and O_3_-present conditions, with the value being substantially larger when O_3_ was present. This was a prerequisite for applying the steady-state method for calculating the OH concentration from the total OH reactivity and the combined OH sources. During the O_3_-free experiment, the dominant OH-reactive chemicals in the chamber were isoprene (from breath) and phenoxyethanol (from lotion), while during the O_3_-present experiment, the dominant OH-reactive compounds were 6-MHO (from skin-oil oxidation), phenoxyethanol, 6-MHO-OH, isoprene, 4-oxopentanal (4-OPA) (from skin-oil oxidation), and geranyl acetone (from skin-oil oxidation) (fig. S2). Phenoxyethanol, a major volatile component of the body lotion, shows maximum values 30 min after the volunteers entered the chamber (30 and 32 ppb for the O_3_-free and O_3_-present experiments, respectively) and then decreases with a reproducible quasilinear trend (Fig. 1B). 6-MHO, the dominant reactive molecule resulting from skin-oil oxidation, reached a maximum (4.6 ppb) during the O_3_ experiment just before volunteers left the chamber (Fig. 1C).

Figure 1D shows the OH concentration calculated from the OH reactivity and volatile organic compound (VOC) measurements using the steady-state method (*11*). With O_3_ present, the average in-room OH concentration generated from four people wearing a body lotion indoors is 1.6 (±0.5) × 10^6^ molecules cm^−3^. Under O_3_-free conditions, the OH concentration was only 1.7 (±0.6) × 10^4^ molecules cm^−3^. Figure 1D also shows the OH concentration when no lotion was worn by the volunteers occupying the chamber (see also table S1 with the experimental results). To investigate this in more detail, kinetic modeling was conducted under the same conditions but with the lotion removed [cyan lines in Fig. 1 (A to D)]. The model results indicate that the application of body lotion causes a substantial increase (~10 to 170%) in OH reactivity (Fig. 1A), which leads to a large decrease (~30 to 140%) in OH concentrations (Fig. 1D). The main cause of the higher OH reactivity is the emission of phenoxyethanol from the lotion, which, in the first few hours, contributes to ~54% of the total reactivity in the presence of O_3_ and to more than 60% in the absence of O_3_. As the emission of phenoxyethanol and its gas-phase concentration decrease over the next 2 hours, the contribution of the lotion to the total OH reactivity decreases to ~25% in the presence of O_3_ and ~40% in the absence of O_3_. The lotion may also affect OH reactivity by affecting the concentrations of other species in the skin. Ozonolysis products of squalene (e.g., 6-MHO and 4-OPA) decrease markedly with the application of the body lotion, as it effectively dilutes the concentration of squalene on the skin surface available for reaction and obstructs the O_3_-skin reaction. In the model, it is assumed that the lotion mixes thoroughly with the skin-oil layer, which originally had a thickness of about 1 μm, resulting in the skin-oil constituents in the much thicker mixed layer being diluted to roughly 10% of their original concentrations. In addition, the body lotion contains linoleic acid, which reacts with O_3_, generating trans-2-nonenal, itself an OH source upon reaction with O_3_.

### Indoor air chemistry of people wearing a fragrance

**Fig. 2.**
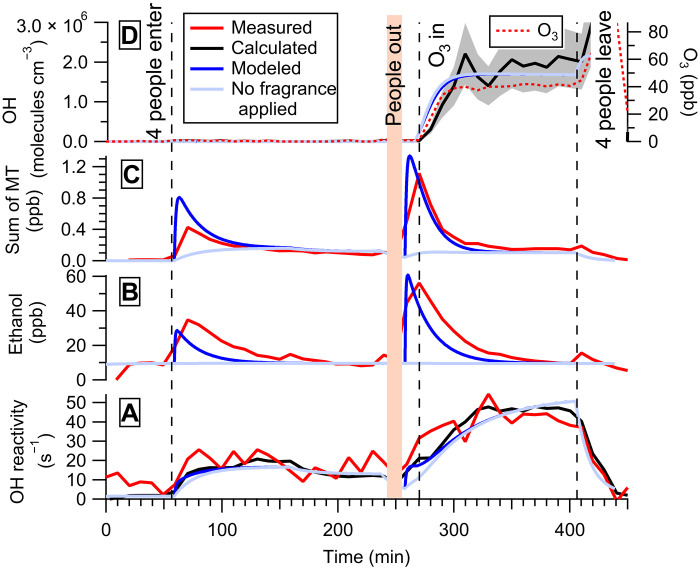
Fragrance case study. In part i of the fragrance case study, four young adult volunteers wearing a complex fragrance, including ethanol as a carrier, occupy the chamber. The fragrance was applied on the back of the hands of two volunteers before entering the chamber in the morning (O_3_-free condition) and before entering the chamber in the afternoon (O_3_ condition). (**A**) OH reactivity. (**B**) Ethanol concentration. (**C**) Sum of monoterpene (MT) concentrations. (**D**) OH and O_3_ concentrations. Red lines represent the measured values, while black lines represent the calculated values, and blue lines represent the values obtained from the kinetic model simulations. The OH concentration was estimated with the steady-state method. Cyan lines are used to indicate the resulting values from the kinetic model simulations for the case when no fragrance is applied. The estimated uncertainties on the measurements are 48% (OH reactivity), 10 to 50% (phenoxyethanol and 6-MHO), 35% (calculated OH concentration; shaded gray area), and 2% (O_3_).

We now examine how perfume applied to the skin affects the chemical composition of the indoor air. Figure 2 shows the measured, calculated, and modeled OH reactivity (per second), along with concentrations of gas-phase species such as the fragrance medium ethanol (in parts per billion), odorous monoterpenes (in parts per billion, here reported as the sum of all monoterpenes), and OH (in molecules per cubic centimeter) generated from four people wearing a popular commercial fragrance inside the chamber (“ck one,” Calvin Klein). The fragrance was uniformly applied to the back of the hand for two volunteers, with one spray per hand dispensed using the fragrance’s built-in vaporizer. The application was made just before (within 5 to 7 min) the volunteers entered the chamber during the morning and was repeated for the afternoon experiments. Experiments were conducted under O_3_-free and O_3_-present (40 ppb) conditions, respectively. In a different experiment, the same volunteers sprayed the same amount of fragrance while inside the chamber. This experiment was used to determine the chemical composition of the fragrance, as discussed in the Supplementary Materials (figs. S3 and S4).

The measured OH reactivity, the calculated OH reactivity from the summed measured gas-phase compounds, and the simulated OH reactivity from the kinetic model yielded similar results, 20 ± 9 s^−1^ (O_3_-free) and 41 ± 16 s^−1^ (O_3_-present) at steady state (Fig. 2A). Figure S5 shows the evolution of the contribution to the OH reactivity of the different gas-phase species measured during this experiment, compared with the benchmark experiment for the same group of volunteers, representing the baseline experiment with no fragrance applied. Among the contributing chemical species, ethanol and monoterpenes show the largest difference between fragrance-free and fragrance experiments. Ethanol contains no double bonds and will act to suppress OH, whereas the monoterpenes (e.g., limonene) are able to both generate OH through reaction with O_3_ and consume OH. Kinetic modeling suggests that under the conditions of this experiment, most of the volatile species present in the fragrances had partitioned to the gas phase before the people entered the chamber. This is reflected by the short e-folding emission times of these species as calculated from table S2. The relatively small increases in the concentrations of these species (Fig. 2, B and C) combined with their rate coefficients did not have a large impact on either the OH reactivity (Fig. 2A) or the OH concentration (Fig. 2D) compared to the simulation where no fragrances were applied. Results were reproducible among replicate experiments with the same group of volunteers [OH reactivity: 41 ± 16 s^−1^ and 45 ± 18 s^−1^; OH: 2.0 (±0.7) × 10^6^ and 2.2 (±0.8) × 10^6^ molecules cm^−3^].

We compared the resulting OH reactivities and concentrations of the molecules common to the lotion experiment and the fragrance experiment in fig. S6. Within the associated uncertainties, the steady-state method yielded comparable OH concentrations for the two different PCPs investigated.

**Fig. 3.**
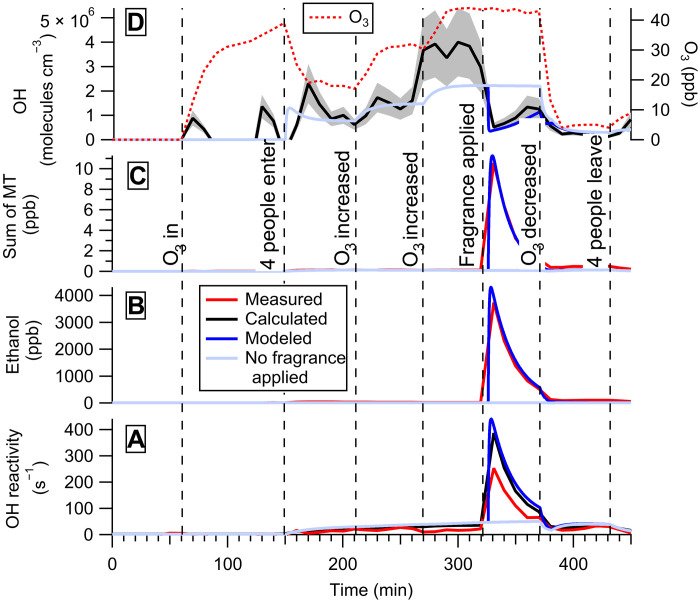
Fragrance case study. In part ii of the fragrance case study, four young adult volunteers wearing a complex fragrance, including ethanol as a carrier, occupy the chamber. In contrast to Fig. 2, the fragrance was applied on the back of the hands of two volunteers while in the chamber. (**A**) OH reactivity. (**B**) Ethanol concentration. (**C**) Sum of monoterpene concentration. (**D**) OH and O_3_ concentrations. Measured values are reported in red, calculated values are reported in black, and simulated values with the kinetic model are reported in blue. The OH concentration was estimated with the steady-state method. The cyan lines indicate the model results for the case of no fragrance being applied during the experiment. The estimated uncertainties on the measurements are 48% (OH reactivity), 10 to 50% (phenoxyethanol and 6-MHO), 35% (calculated OH concentration; shaded gray area), and 2% (O_3_). “O_3_ in” indicates the start of O_3_ generation, while “O_3_ decreased” indicates the end of O_3_ generation.

Figure 3 shows the measured, calculated, and modeled OH reactivity with the OH concentration calculated from the measurements and simulated with the kinetic model and measurements of gas-phase species in the presence of people who applied the same amount of fragrance while in the chamber. Very high concentrations of ethanol (3740 ppb) and monoterpenes (10.6 ppb) are produced shortly after the fragrance is applied, leading to maximum values of OH reactivity between 253 s^−1^ (measured) and 387 s^−1^ (calculated). This difference can be explained by the OH reactivity instrument acquisition time, which averages over the short intense spike captured by the VOC measurements. The largest contributors to the OH reactivity at the time the fragrance is applied in the chamber (335 min) are ethanol (76%), the sum of monoterpenes (11%), 6-MHO (5.2%), acetaldehyde (3%), isoprene (3%), and 4-OPA (1.3%) (fig. S7).

The large increase in OH reactivity leads to a significant decrease in OH concentration (86% loss from the inferred concentrations at the point the fragrance is applied; Fig. 3D). As expected from previous studies (*4*, *7*, *8*), an increase in the indoor O_3_ concentration when people occupy the chamber leads to increasing concentrations of geranyl acetone, 4-OPA, and 6-MHO (fig. S8), which leads to steadily increasing OH concentrations up to a maximum value of 3.6 (±1.3) × 10^6^ molecules cm^−3^ when O_3_ was 44 ppb. The removal of species emitted by the fragrances from the chamber is mainly controlled by indoor-to-outdoor mass transport with an air exchange rate of 2.9 hour^−1^ during the experiments, leading to a factor of 3 decrease in OH reactivity over a period of 30 min.

We simulated the case of people wearing both the lotion and the fragrance with the KM-SUB-Skin-Clothing model, assuming that the people in the chamber also sprayed the fragrance on themselves immediately upon entering the chamber. For comparison purposes, we also simulated the same scenario with people applying only the lotion, only the fragrance, and no lotions or fragrances. The results show that in the early stages, the fragrance causes an elevated OH reactivity and a significant decrease in OH concentrations, while at longer times, the lotion is more important in controlling the OH reactivity and concentration (fig. S9). This is expected as organic compounds in the fragrances, such as ethanol, rapidly partition into the gas phase and are quickly removed by air exchange, while phenoxyethanol partitions out of the lotion at a slower rate and remains at an elevated concentration in the gas phase for a longer time.

### Indoor air chemistry of a single-component fragrance

**Fig. 4.**
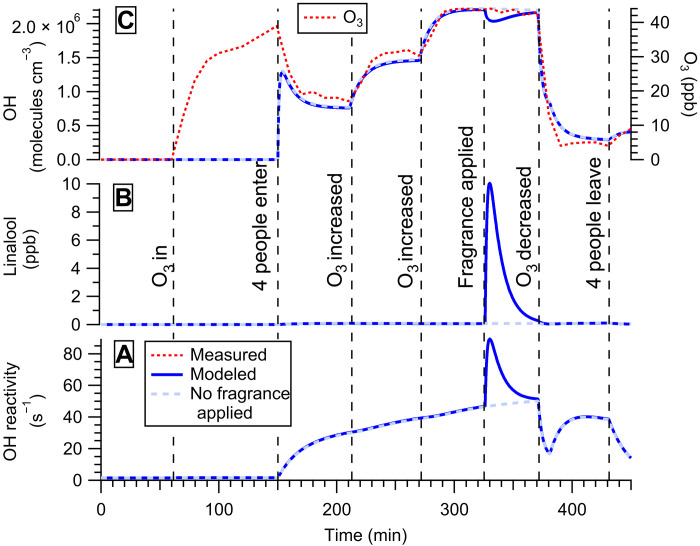
Fragrance case study. In part iii of the fragrance case study, sensitivity simulations performed using the KM-SUB-Skin-Clothing model showing the impact of a fragrance containing only linalool on (**A**) OH reactivity, (**B**) linalool concentrations, and (**C**) OH concentrations. The red dotted line represents the measured O_3_ concentration during the fragrance experiment. The blue line and the dashed cyan line represent the results from the sensitivity simulations, with and without fragrance, respectively. “O_3_ in” indicates the start of O_3_ generation, while “O_3_ decreased” indicates the end of O_3_ generation.

We have shown that fragrances having ethanol as a carrier influence air chemistry by depleting OH (Figs. 2 and 3). A sensitivity test was conducted using the kinetic model with the same conditions as in Fig. 3, including the same O_3_ concentration that was measured during the Fig. 3 experiment, but with linalool, a terpene alcohol present in lavender (*31*), being the only compound that was emitted from the fragrance (i.e., no ethanol carrier) and its emission rate being adjusted to obtain a maximum concentration of 10 ppb (Fig. 4). The OH reactivity increased significantly when linalool was emitted into the gas phase (Fig. 4), as OH is highly reactive toward linalool (*32*). However, OH concentrations decreased by less than 10%, as the reaction of linalool with O_3_ produces OH efficiently (Fig. 4). This sensitivity test demonstrates that the ozonolysis of linalool, in the absence of ethanol, may not have a large impact on the OH concentration inside a room. Note that the formation of organic products due to the oxidation of linalool did not substantially affect the OH concentration or reactivity under the conditions of the experiment due to their relatively low concentrations and lower OH reaction rate coefficients (table S5).

### How lotions and fragrances affect the human oxidation field

**Fig. 5.**
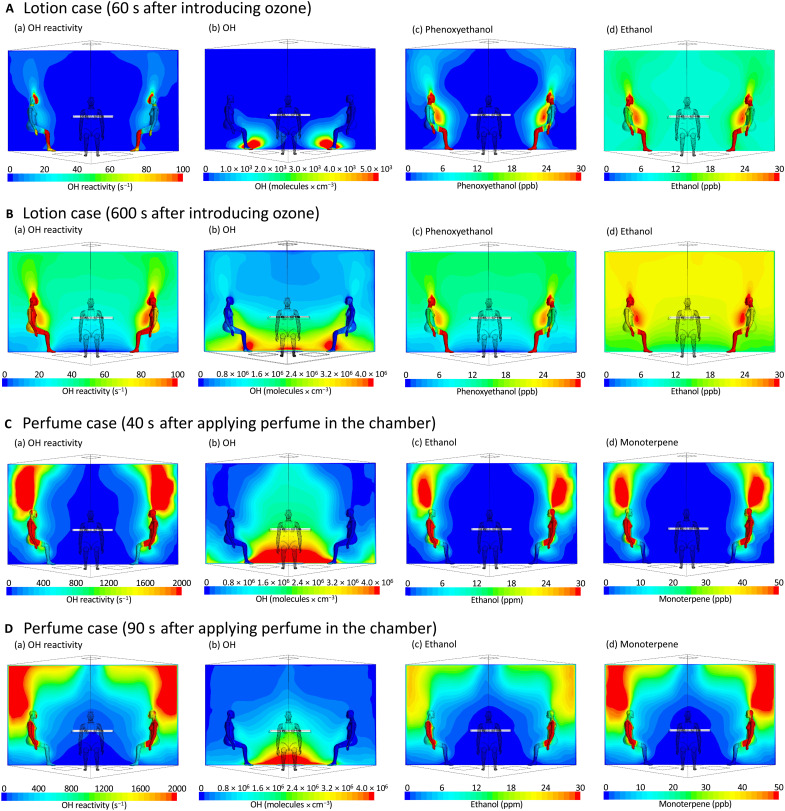
Distribution of the human OH field when a lotion or a fragrance is applied in the room. Human OH field with lotion (**A** and **B**) and fragrance (**C** and **D**). Spatial distributions of OH reactivity and gas-phase species under two conditions: (A) and (B) represent the scenario where lotion was applied to the exposed skin of four occupants, observed at 60 and 600 s, respectively, after O_3_ was introduced into the room from a perforated inlet on the floor. (C) and (D) depict the scenario where a fragrance was applied to the back of the hands of two occupants sitting in the chamber, observed at 40 and 90 s, respectively, after the fragrance application.

We now examine the influence of the aforementioned emissions on the human oxidation field. Figure 5 (A and B) illustrates spatial distributions from simulations conducted at 60 and 600 s after introducing O_3_ into the room of (a) OH reactivity and gas-phase species: (b) OH radical, (c) phenoxyethanol, and (d) ethanol as computed by computational fluid dynamics (CFD) simulations when individuals apply lotion to uncovered skin. Following the lotion application, species such as phenoxyethanol and ethanol were released. These species exhibited an upward movement, following the buoyancy-driven thermal plume around the occupants and subsequently dispersing into the ambient air. These compounds continued to be released into the ambient air, with their concentrations steadily rising even 10 min after the lotion application. Their concentration near the chest area was determined to be 1.6 times higher, and near the nose 2.8 times higher, than those in the ambient air. As O_3_ was supplied from the perforated inlet located at the floor and reacted with the skin lipids on the human skin surface, carbonyls generated by the ozonolysis of squalene were released from the human surface and dispersed to the ambient air. Ozonolysis products and lotion-emitted compounds from the human surface became the predominant contributors to OH reactivity. The mean OH reactivity determined in the room increased from 2.5 s^−1^ at 60 s to 36.6 s^−1^ at 600 s after introducing O_3_, while the OH reactivities determined near the chest reached 21.2 and 82.1 s^−1^, respectively. The mean OH radical concentration in the room at 600 s after the introduction of O_3_ was 1.2 × 10^6^ molecules cm^−3^ following a strong spatial gradient with a maximum near the floor, where the O_3_ inlet is located.

Figure 5 (C and D) shows the simulations for conditions at 40 and 90 s after the fragrance was applied on the back of the hands of two occupants while sitting in the room. Similar to the lotion case, the species moved upward due to the occupants’ thermal plumes. In contrast to the lotion case, the emission rates of the fragrance components such as ethanol and monoterpenes were considerably faster than those of the lotion components, resulting in a rapid concentration increase in both ethanol and monoterpenes above the occupants’ heads. Ethanol and acetaldehyde concentrations peaked 40 s after applying the fragrance, while monoterpene concentrations reached their peak at 90 s (fig. S11). Notably, at the peak concentrations of these compounds, the levels above the head were found to be up to 10 times higher than the levels in the surrounding ambient air. Ethanol and monoterpenes from the fragrance were the main contributors to the OH reactivity, resulting in mean room OH reactivity values of 303 s^−1^ at 40 s and 438 s^−1^ at 90 s, while values exceeding 2000 s^−1^ were obtained above the head of the occupants. Conversely, OH concentrations were lower in areas where concentrations of ethanol and monoterpenes were larger, as OH radicals were consumed through reactions with these chemicals.

## DISCUSSION

This study has determined that the human oxidation field generated by people exposed to O_3_ indoors is substantially disrupted when PCPs are worn. Specifically, we experimentally and numerically investigated two cases: people wearing a body lotion and people wearing a fragrance. Body lotion emissions (such as phenoxyethanol) react with OH, while the reduced availability of skin lipids exposed to O_3_ affects the emission of skin-oil oxidation compounds such as 6-MHO. Considering the interplay between the OH production rate and the OH loss rate (i.e., reactivity), the resulting OH concentration at steady state is substantially reduced by both the (i) limited OH production from skin emissions and (ii) enhanced OH reactivity due to the lotion emissions. The 6-MHO concentration measured in the occupied chamber under steady-state conditions in the experiment when volunteers were wearing the body lotion with respect to the benchmark experiment was 34% lower. We also investigated a widely used unisex fragrance with a notable citrus scent. When applied at typical levels, we would expect that the scent itself (a mix of monoterpene compounds dominated by limonene, β-pinene, and γ-terpinene) is able to increase the OH production rate resulting from human skin emissions. However, the fragrance composition was dominated by ethanol, whose concentration reached almost 4 parts per million (ppm) at the time the fragrance was applied (sprayed) inside the room (while the sum of monoterpenes reached about 11 ppb). Ethanol concentration measured inside the chamber when the fragrance was applied by the volunteers with respect to the summed concentration of terpenes and ethanol was >99%. For comparison, the ethanol concentration measured from breath exhalations of people consuming beers in a crowded soccer stadium during a Mainz match reached a maximum of 400 ppb (*33*). Ethanol has a moderate impact on total OH reactivity when present in low concentration. The effect here is remarkable due to the large concentration of ethanol inside the room shortly after the fragrance is applied. The overall effect of a scent diluted in ethanol (i.e., any commercial fragrance) is therefore a reduction of the human-generated OH field due to the much larger indoor OH loss rate with ethanol compared with the OH production rate from the scented terpenes. The application of a fragrance and a lotion together showed that fragrances affect the OH reactivity and concentration at shorter times, whereas lotions show more persistent effects, consistent with the rate of emissions of organic compounds from these PCPs. We also simulated the case of people applying pure linalool, to represent the case of a fragrance not diluted in ethanol (i.e., a pure essential oil). Linalool is a terpene alcohol that reacts rapidly with O_3_ and OH under the conditions of the experiment, but its net effect on the OH field generated from human emissions indoors was less than 10%.

Experimental tests with one fragrance and one body lotion illustrated possible chemical processes occurring in indoor air when people wear these products. While thousands of different fragrances and lotions exist on the market, there are some general conclusions valid for any product that can be drawn on the basis of our tests. Fragrances are classified into perfumes, eau de perfume, eau de toilette, and eau de cologne depending on the percentage of odorous chemical species of natural or synthetic origin dissolved in a solvent, which is usually ethanol; perfumes have the strongest odor and lasting effect, with a more concentrated percentage of odorous compounds, between 15 and 30% (*34*). However, only 34% of consumers globally prefer perfumes over more diluted fragrances (*35*); consequently, ethanol is by far the dominant molecule emitted into an indoor environment when a fragrance is applied. For this reason, in the real world, an applied fragrance indoors would be expected to suppress the personal human oxidation field. In contrast with fragrances, lotions have more variable compositions, typically including a number of unsaturated fatty acids. Despite their variable composition, we expect most lotions to suppress the human oxidation field due to a combination of dilution of skin-oil constituents and reduced interaction between O_3_ and the skin. In addition, marketed lotions contain preservatives acting as antimicrobial agents. One of the most tolerated and widely used is phenoxyethanol (*36*, *37*), which further contributes to suppressing the human oxidation field by reacting with the OH radicals as experimentally demonstrated in this study.

The indoor OH concentration significantly affects air chemistry, the quantity, and type of secondary species produced, and accordingly overall human exposure to these chemicals. Hydroxy radicals are derived from O_3_-initiated chemistry and can be counted among the “O_3_ reaction products.” The net concentration of these O_3_ reaction products scales with the difference between outdoor and indoor O_3_ concentrations (*38*). He *et al.* (*39*) examined associations of O_3_ reaction products with biomarkers of cardiorespiratory pathophysiology that had been measured in two prior studies—one with asthmatic children and one with healthy adults. Using biomarkers from the children with asthma, He *et al.* (*39*) found that “O_3_ loss exposure (but not O_3_ exposure) was significantly and adversely associated with fractional exhaled nitric oxide (airway inflammation), Z_5_ (airway impedance at 5 Hz), X_5_ (airway reactance at 5 Hz), Fres (airway reactance; resonant frequency), and FEV1/FVC (airflow obstruction risk).” Using biomarkers from the healthy adult study, they found “significant and adverse effects of O_3_ loss exposure (but not O_3_ exposure) on nitrate and nitrite in exhaled breath condensate (pulmonary oxidative stress), fractional exhaled nitric oxide, 8-OHdG (systemic oxidative stress), 20-HETE (vasoconstriction), diastolic blood pressure, and systolic blood pressure.” Compared to O_3_, OH is a less discriminating oxidant and reacts faster with most organic compounds found in indoor environments. Increased OH concentrations result in increased oxygen-to-carbon ratios among indoor pollutants. The oxidized compounds have larger water solubilities (*40*, *41*) and hence larger lung fluid/air partition coefficients. The increased partitioning into lung fluid, compared to precursors, results in longer residence times in the lung and increased opportunities for the oxidized pollutants to interact with cells.

Hydroxyl radicals affect not only airborne pollutants but also pollutants on indoor surfaces. OH is a key player in autoxidation reactions that occur on indoor surfaces both directly and via the alkylperoxy radicals formed when OH abstracts a hydrogen from an organic reactant. This has been demonstrated in recent experiments showing multiple products of di-2-ethylhexyl phthalate formed in indoor dust and surface films as a consequence of OH radical reactions (*42*). Such products can be inhaled with resuspended dust, dermally absorbed via contact with surfaces, or ingested via dust transferred to the hands and then the mouth. The latter is especially noteworthy for infants.

In some instances, the chemical transformations resulting from increased OH concentrations may yield products that have reduced toxicities compared to their precursors. However, the net consequence of increased OH levels appears to be less healthy indoor environments. That said, a more comprehensive assessment of the relative toxicities of the associated precursors and products is required before making a recommendation. The best course of action may depend on the specific conditions of the individual indoor environment. While the application of PCPs indoors, as shown in our study, limits the potential for the production of harmful unregulated products in the indoor environment, direct exposure to chemicals contained in PCPs should still be considered. At the same time, recent research has shown that volatile chemical products, including PCPs, can escape our homes, influencing O_3_ formation outside and deteriorating the air quality in densely populated urban areas (*29*, *30*).

Oxidation reactions lead to the formation of condensable vapors, leading to the growth of suspended particles, which can penetrate the human body and deposit in the lung. Thereby, the generated secondary products can be effectively carried by particles deep into the human body. Direct nanocluster aerosol formation (<3 nm) from skin-oil ozonolysis has been reported (*9*, *43*, *44*). The model simulations conducted in this work highlight many chemical species that accumulate in larger concentrations in the vicinity of the breathing zone. To evaluate the health effects of chemical sources indoors, it is important to know the identity and toxicity of the products evolving from the chemistry associated with these processes. The exact compounds present in the breathing zone will depend on our own oxidation field and how we perturb it with PCPs. Alteration of the personal OH field in this way can also affect the overall quality of life as perceived through the olfactory pathway (*45*).

## MATERIALS AND METHODS

### Experimental design

The experiments described here were conducted in a 22.5-m^3^ stainless steel climate chamber at the Technical University of Denmark (DTU) within the framework of the ICHEAR 2 project. The ICHEAR 2 project was a continuation of ICHEAR, which aimed at characterizing baseline human emissions under several different environmental conditions. The focus of this study is given to the indoor air chemistry resulting from people applying PCPs, specifically fragrances and body lotions, indoors. In all the considered experiments, four young adult males (19 to 27 years old; body mass index: 20.7 to 26.1) volunteered to sit in the chamber wearing shorts and shirts with long sleeves while being exposed to moderate temperature (~26°C), low relative humidity (RH; ~25%), and O_3_ (0 ppb or steady state in the occupied chamber at 38 to 45 ppb). PCPs were investigated in two sets of experiments with two different groups of volunteers for the application of a body lotion (set 1) and for the use of a fragrance (set 2). Set 1 involved the benchmark condition (no lotion applied) and the application of a lotion. A commercial lotion composed of aqua, glycerin, *Brassica campestris* seed oil, *Butyrospermum parkii* butter, ceteareth-12, ceteareth-20, cetearyl alcohol, ethylhexyl stearate, *Simmondsia chinensis* seed oil, tocopherol, caprylyl glycol, citric acid, sodium hydroxide, acrylates/C10-30 alkyl acrylate crosspolymer, sodium gluconate, and phenoxyethanol was chosen for this experiment. *B. campestris* seed oil (rapeseed oil), *B. parkii* butter (shea butter), and *S. chinensis* seed oil (jojoba oil) are common lotion ingredients composed of fatty acids, present in the dominant fraction in their saturated and monounsaturated forms (therefore with less double bonds available for reacting with O_3_ compared to skin-oil squalene) with variable relative composition depending on their origin. Subjects applied, on average, 5.9 g of lotion per person on a surface area of 0.443 m^2^ including the forearms, hands, lower legs, and face/neck [calculated based on an average body surface area of 1.93 m^3^ of the participating subjects and the percentage of total body surface area per body part taken from the US Environmental Protection Agency (EPA) Exposure Factors Handbook (*46*)] before entering the chamber in the morning (O_3_-free condition) and before entering the chamber in the afternoon (O_3_ condition).

A popular commercial fragrance described to release top notes of bergamot, lemon, mandarin, fresh pineapple, papaya, cardamom, and green tree accord; heart notes of florals such as violet, rose, and lily of the valley; and bottom notes of green tea, oak moss, cedar wood, and sandalwood, among others was chosen for the fragrance experiments. The fragrance experiments consisted of one benchmark experiment with no fragrance applied, two replicate experiments where fragrance was applied before volunteers entered the chamber in the morning (O_3_-free condition) and before they entered the chamber in the afternoon (O_3_ condition), and one experiment where volunteers applied the fragrance while in the chamber with varying O_3_ concentration. The fragrance was applied by spraying the fragrance built-in vaporizer once on the back of the hands of two volunteers. The chamber was furnished with five tables, four chairs, and two mixing fans. The same protocol used for ICHEAR was adopted for the ICHEAR 2 experiments (*5*). The trace gas concentrations and OH reactivity of the unoccupied chamber were measured before each experiment to determine the background. The chamber was ventilated with outdoor air, which was filtered with activated carbon filters to remove O_3_ and particle filters. The air was supplied to the chamber through a perforated floor with an air change rate of 2.9 hour^−1^. Inside the chamber, air mixing was achieved by using two fans directed at the walls of the chamber. O_3_ was generated in the HVAC system downstream of the activated carbon filter. Pure oxygen was delivered through a Jelight 600 ultraviolet (UV) O_3_ generator (Jelight Company Inc., USA). O_3_ was added with the supply air with a mixing ratio at the inlet of ~100 ppb, which resulted in a chamber level of ~40 ppb when four people were present. Volunteers showered the night before the experiments with provided fragrance-free liquid soap and shampoo. On the day of the experiment, they wore a set of provided identical new clothes laundered with fragrance-free laundry detergent and tumble dried before the experiment. On the day of the experiment, they entered the O_3_-free chamber at 0930, left for a short lunch break (provided with bread, butter, and sliced cheese) between ~1230 and 1245, and entered again the chamber for the afternoon experiment with O_3_ until 1515, when the volunteers left the chamber. O_3_ generation began 10 min after the volunteers returned into the chamber after the lunch break.

### Trace gases and OH reactivity measurements

VOCs were measured online using a proton transfer reaction–time-of-flight–mass spectrometer (PTR-TOF-MS 8000, IONICON Analytik GmbH, Austria), a custom-built fast gas chromatography–mass spectrometry (GC-MS) instrument, and offline using a GC-TOF-MS instrument (Markes International, UK) with two-bed custom-made sorbent tubes made of Carbograph 1 and 5. The PTR-TOF-MS was operated under standard conditions [drift pressure (*P*_drift_) = 2.2 mbar, inlet temperature (*T*_inlet_) = 60°C, and *E/N* = 137 Townsend (Td)] with a mass resolution of 4000 at 96 amu and a time resolution of 20 s. 4-OPA, 6-MHO, and 17 other compounds [methanol, acetonitrile, acetaldehyde, ethanol, acrylonitrile, acetone, dimethyl sulfide, isoprene, methyl vinyl ketone (MVK), methyl ethyl ketone, benzene, xylene, 1,3,5-trimethylbenzene, α-pinene, caryophyllene, octamethylcyclotetrasiloxane, and decamethylcyclopentasiloxane] were calibrated using certified gas standard mixtures (Westfalen AG, Germany, and Apel-Riemer Environmental Inc., USA). The mixing ratios of the masses of the compounds not included in the gas standards were calculated using the theoretical method (*47*, *48*) where the mass-dependent transmission efficiency was derived from calibration results, and a constant proton transfer reaction rate coefficient of 2.5 × 10^−9^ cm^3^ molecule^−1^ s^−1^ was used. The total uncertainties on the concentrations of the calibrated species ranged from 5 to 23%, while the total uncertainties for other species concentrations were up to 50%. The uncertainties for these theoretically calculated species arise from their exact proton transfer reaction rate coefficients and potential fragmentation fractions.

The custom-built fast GC-MS (*49*) uses a three-step cryogenic preconcentration system before GC separation and detection with a quadrupole mass spectrometer (QMS) operating in selected ion monitoring (SIM) mode. In this project, a sample volume of 20 to 40 ml of air was collected. The time resolution of the system was 3 min. A certified gravimetrically determined gas standard mixture including isoprene and propanal (Apel-Riemer Environmental Inc., USA) was used to calibrate the fast GC-MS every day during the campaign. To ensure interference-free measurements, the fast GC-MS system was equipped with a sodium thiosulfate O_3_ scrubber upstream of its inlet line. This filter scrubber was exchanged at latest every 5 days, to assure proper O_3_ scrubbing (*50*). The overall measurement uncertainty of the system was <20%.

Two-liter air samples were collected on two-bed custom-made sorbent tubes made of Carbograph 1 and 5 equipped with a sodium thiosulfate O_3_ scrubber upstream of the sampling line for offline analysis with a GC-TOF-MS. The GC-TOF-MS was coupled with a thermodesorption unit to desorb the analytes from the sampling tubes using two sequential stages, both performed at 250°C for 10 min. The GC column was a dimethyl tris-buffered saline β-cyclodextrin–based column (0.25 μm, 0.25 mm inside diameter, 30 m/liter; MEGA, Italy), which separates the analytes according to the boiling point and enantiomeric configuration. The separation method was specifically designed for the separation of chiral monoterpenes (C_10_H_16_) and sesquiterpenes (C_15_H_24_) and consists of an initial 5 min when the oven temperature was held at 40°C, after which it was increased at a rate of 1.5°C/min from 40° to 150°C and then increased further at a rate of 30°C/min from 150° to 200°C. Detection was performed using a TOF-MS, which fragments the analytes through electron impact ionization at −70 eV for quantification and identification of the chemical species. Identification of the main chemical compounds was obtained by comparing the MS spectra with the MS library for the same ionization energy (National Institute of Standards and Technology library), by injection of a calibration gas standard mixture (19 biogenic VOCs provided by Apel-Riemer Environmental Inc., USA) and by the use of liquid standards. Chromatogram peak areas were integrated through TOF-DS software (Markes International, UK). The standard gas used is a gravimetrically certified concentrated mixture (~100 parts per billion by volume) of 19 biogenic volatile organic compounds (BVOCs) including, among other species, four couples of chiral terpenoids (α-pinene, β-pinene, limonene, and linalool), isoprene, and one sesquiterpene (β-cayophyllene). The GC-TOF sensitivity was quantified by measuring the concentration of BVOCs in standard sorbent tubes filled with 2 liters of gas standard diluted in a pure synthetic air flow. The obtained calibration factors were confirmed with standard cartridges containing a known BVOC concentration analyzed in a workflow, whereas for every five samples analyzed, a standard cartridge was also measured. The standard cartridges analyzed in this way were used to determine the precision of the analysis (including any drift from the mass spectrometer), which was quantified as 22% [for additional details, refer to (*51*–*53*)].

Total OH reactivity was measured using a custom-built comparative reactivity method instrument, which consists of a glass flow reactor coupled to a PTR-QMS (IONICON Analytik GmbH, Austria). The PTR-QMS was used to monitor the concentration of a reference molecule (pyrrole, C_4_H_5_NH^+^, mass/charge ratio 68) competing with trace gas molecules in air for reaction with in situ–generated OH radicals. Hydroxyl radicals were generated inside the reactor from the photolysis of water vapor, which was achieved by using a Hg UV lamp (emitting at 184.9 nm) and wet N_2_. Pyrrole (Westfalen AG, Germany) was measured in clean air and dry N_2_ after photolysis (concentration C1), in the presence of OH (C2), and in ambient air (C3). Assuming pseudo–first-order kinetics inside the reactor ([pyrrole] >> [OH]), the OH reactivity is obtained from pyrrole concentrations C1, C2, and C3 with the following Eq. 1

$$R_{\text{air}}=\frac{\left(C3-C2\right)}{\left(C1-C3\right)}\times k_{\text{pyrrole}+\text{OH}}\times C1$$ (1)

where *k*_pyrrole+OH_ is the rate constant of the reaction between pyrrole and OH (1.20 ± 0.16) × 10^−10^ cm^3^ molecule^−1^ s^−1^. The PTR-MS was operated under standard conditions (*P*_drift_ = 2.2 mbar, *E/N* = 130 Td, and *T*_inlet_ = 60°C), C1 was quantified by using an OH scavenger, and switches between C2 and C3 were programmed to occur every 5 min. The data workflow includes PTR-MS calibration with pyrrole at different levels of humidity, humidity correction on C2 to correct for OH recycling when humidity changes, reactivity calibration for deviation from pseudo–first-order kinetics with test gases *i* having different *k*_*i+*OH_, and dilution of the sampling flow into the flow reactor. All correction factors were determined experimentally, and test gases included isoprene and α-pinene. The resolution of the OH reactivity measurement was 1 to 10 min, the limit of detection (1σ) is ~5 s^−1^, and the quantified total uncertainty is up to ~48%. Detailed information on the measurements can be found in (*7*, *8*, *11*) and references therein.

A common inlet of fluorinated ethylene propylene [with an outer diameter of ^1^/_2_ inch (1.27 cm) and a length of approximately 5 m] was used to draw the air (inlet flow of ~13 liters min^−1^) from the chamber outlet toward the measurement devices. From this common inlet line, individual substreams were sampled by each instrument. Additional data used in this study include NO*_x_*, O_3_, temperature, and humidity that were all measured through separate inlets. Nitrogen monoxide (NO) was measured with a fast response chemiluminescence detector (CLD) 780 TR (ECO PHYSICS AG, Dürnten, Switzerland). The chemiluminescence technique for NO measurement involves the reaction of NO with O_3_ in a reaction chamber at low pressure to produce excited nitrogen dioxide (NO_2_), which then emits a photon, as it returns to the ground state. The intensity of the light emitted is directly proportional to the concentration of NO in the sample, which is measured by a photomultiplier. More details about the measurement technique can be found in Drummond *et al.* (*54*). A NO_2_-specific photolytic converter, Teledyne blue light converter (BLC) (Teledyne API, California, USA), was used to measure NO_2_ by converting NO_2_ in the air sample into NO. The photolytic converter was placed in line with the air sample and would be switched on and off sequentially. This way, we could measure NO_2_ by subtracting the measurement of NO with the converter off from the measurement with the converter on. The conversion efficiency of the BLC was determined using NO primary standard gas from the National Physical Laboratory (Teddington, UK) and a Thermo Environmental TEI146C (Massachusetts, USA) gas-phase calibrator and titrator. The same system was used to calibrate the CLD 780 TR. Air was sampled at a flow rate of 3.5 liters min^−1^ after passing through a 5-μm polytetrafluoroethylene filter. Data from the CLD were sampled every 10 s, and a CR3000 data logger (Campbell Scientific, Logan, Utah, USA) was used to collect data and to control the switching on and off of the converter. The limit of detection using 3σ was 230 ppt for NO, 260 ppt for NO*_x_*, and 540 ppt for NO_2_. Corresponding uncertainties were 80 parts per trillion (ppt) for NO, 90 ppt for NO*_x_*, and 240 ppt for NO_2_.

O_3_ was monitored with a 2B Technologies Model 205 O_3_ monitor (USA) with a time resolution of 10 s (accuracy: 1.0 ppb or 2% of reading). Air temperature and RH were monitored with a Vaisala GMW90 (accuracy: temperature ±0.5°C, RH ±2.5% below 60%, and CO_2_ ± 30 ppm + 2% of reading; Vaisala Corporation, Finland) connected to a HOBO UX120-006M four-channel analog data logger (Onset Computer Corporation, USA) with a time resolution of 1 min.

### OH reactivity calculated from the measured VOCs

The calculated OH reactivity was determined from the measured VOCs using Eq. 2

$$R_{\text{calculated}}=\sum_{i}k_{i+\text{OH}}\times \left[{\text{VOC}}_{\text{OH}\_i}\right]$$ (2)

where *k*_(*i*+OH)_ is the rate constant for the reaction between the *i*th VOC and OH and [VOC_OH_*i*_] stands for all VOCs reactive to OH concentration.

### Calculated OH radical concentration

The hydroxyl radical (OH) concentration was determined with Eq. 3 using the “steady-state method” described in (*11*). Such method assumes that the production rate and loss rate of the OH radicals are in balance, which is a reasonable assumption given the short residence time of the short-lived species measured in the experiments. Direct measurements of individual VOCs, O_3_, and total OH reactivity from the experiments described above were used to determine the OH production and loss rates, respectively

$${\left[\text{OH}\right]}_{\mathrm{ss}}=\frac{\sum_{i}{\text{yield}}_{i}\times k_{i+O3}\times \left[{\text{VOC}}_{O3\_i}\right]\times \left[O_{3}\right]}{\sum_{i}k_{i+\text{OH}}\times \left[{\text{VOC}}_{\text{OH}\_i}\right]}$$ (3)

where yield*_i_* is the fractional OH yield from the ozonolysis reaction between compound *i* and O_3_, *k*_(*i*+O3)_ is the rate constant of the reaction between alkenes and O_3_, VOC_O3_*i*_ stands for all VOCs reactive to O_3_ and here refers to the measured hydrocarbons with unsaturated (C═C) bonds, O_3_ is the measured O_3_ concentration, *k*_(*i*+OH)_ is the rate constant for the reaction between the *i*th VOC and OH, and VOC_OH_*i*_ stands for all VOCs reactive to OH. The term in the numerator represents the total OH production rate from alkene ozonolysis reactions, and the term in the denominator represents the total OH loss rate, which we have determined directly as the total OH reactivity. Possible OH recycling from fragrances (*55*) and specifically from monoterpenes (*56*–*59*) is accounted for in the measured concentrations of the alkene compounds and the measured OH reactivity. The measured alkenes considered in Eq. 3 were isoprene, limonene, 6-MHO, OH-6-MHO, geranyl acetone, MVK, methacrolein, 4-methyl-8-oxo-4-nonenal (4-MON), 4-methyl-4-oc-tene-1,8-dial (4-MOD), and trans-2-nonenal.

### Kinetic modeling

Figure S13 shows the kinetic multilayer model of the surface and the bulk chemistry of the skin and clothing (KM-SUB-Skin-Clothing) (*11*, *60*), which has been adapted to include both uncovered skin and skin covered by clothing in this study. The gas phase above the uncovered and covered skin is shared but all other layers are treated separately. The model layers include the gas phase, clothing, gap between the clothing and skin, skin oil, stratum corneum, viable epidermis, and blood vessels. Mass transport and chemical reactions are resolved in the gas phase, boundary layer, clothing, gap between the clothing and skin, and all skin layers. The reaction of O_3_ with squalene is treated in the model forming a range of products: acetone, 6-MHO, geranyl acetone, 4-OPA, 4-MON, and 4-MOD. The reaction of O_3_ with other skin lipids forms various products (butanal, acetaldehyde, propanal, and trans-2-nonenal), which are modeled by assuming a yield from this generic reaction (*61*). Background, breath, and skin emissions of compounds are also included in the model using emission rates, which were obtained by fitting to measurements performed in the presence and absence of people (table S3).

Species originating from fragrances and lotions are treated using emission rates, which vary over time, as the species evaporate from or partition out of the fragrance or lotion and were determined by fitting to measurements (table S2). Lotions also affect the reactivity of the skin-oil layer as follows. The average amount of lotion applied per person to uncovered skin with an average surface area of 0.4425 m^2^ was 5.9 g. Two people had uncovered forearms with an average total uncovered skin surface area of 0.516 m^2^, and two people had covered forearms with an average uncovered skin surface area of 0.369 m^2^ to which the lotion was applied. Assuming a density of 1 g cm^−3^ for the lotion, this results in a lotion thickness of approximately 13 μm. In the model, we estimated the skin-oil constituents in the much thicker mixed layer being diluted to ~7% of their original concentrations. The lotion also contains species that can react with O_3_ such as unsaturated fatty acids (e.g., in *B. campestris*). *B. campestris* contains linoleic acid, which reacts with O_3_ to form trans-2-nonenal (*62*, *63*). We treat the reaction of O_3_ with these species using an effective first-order rate coefficient and a yield of trans-2-nonenal (table S4). Most parameters used in the model have been kept identical to our previous work (*11*, *60*), but previously unknown and updated parameters are summarized in table S4. All gas-phase chemical reactions are summarized in table S5. The KM-SUB-Skin-Clothing model was used to determine the most important species, reactions, and emission rates with regard to OH concentrations and reactivity to include in CFD simulations (tables S2, S3, and S5). It was also used to output O_3_ uptake coefficients and product yields, and for simplicity, these were averaged over the time when O_3_ reached a constant concentration for implementation into the CFD simulations (table S6). For “no fragrance” and “no lotion” simulations, the emission rates in table S2 were not included in the model. In addition, for no-lotion experiments, the skin-oil constituents were not diluted, and the first-order production rate of trans-2-nonenal described above was not included in the model. The no-fragrance and no-lotion simulations enable the effect of parameters associated with fragrances and lotions to be investigated, while keeping all other environmental, skin, and clothing parameters constant, which is difficult to achieve experimentally.

### CFD modeling

The transient CFD modeling framework was developed to simulate the transport and chemical reactions associated with skincare products. These reactions encompass O_3_ interactions with human surfaces wearing skincare products and subsequent gas-phase reactions of oxidants (O_3_ and OH radicals) with ozonolysis products. The CFD domain replicated experimental conditions, including room geometry, occupancy, and supply O_3_ concentrations (see fig. S14). The simulated volume of the room is 28.5 m^3^, with four-seated human geometries positioned at each corner. O_3_-laden air was introduced through five floor supply inlets and exhausted through the ceiling outlet. Manikin surface temperatures were maintained at 35°C, while the average room air temperature was set at 25°C, creating a convective thermal plume around occupants due to the temperature gradient between human surfaces and ambient air. Uncovered skin is depicted in magenta in fig. S14. Fragrance was applied to the hands of two occupants, while lotions were applied to the uncovered skin of all occupants.

The CFD model simulated that occupants continuously exhaled isoprene and NO at a constant mass flow rate. Furthermore, human surfaces emitted chemical species, including ethanol, monoterpene, and linalool, even when individuals were not wearing PCPs. Indoor surfaces also generated ethanol, acetaldehyde, and NO. The room average concentrations were maintained under steady-state conditions, with 3.1 ppb of isoprene, 0.8 ppb of NO, 8 ppb of ethanol, and 1.7 ppb of acetaldehyde.

The KM-SUB-Skin-Clothing model provided uptake coefficients for ozone and yields of ozonolysis products on the human surface. Using these values, deposition rates of ozone and emission rates of squalene ozonolysis products on the human surface were computed. The CFD model incorporated a total of 27 chemical reactions for the fragrance case and 24 reactions for the lotion case. Ozone reactions with human occupants generated primary and secondary ozonolysis products (e.g., 6-MHO, 4-OPA, geranyl acetone, 4-MON, 4-MOD, and trans-2-nonenal). These products underwent further reactions with ozone, resulting in the production of OH radicals and secondary products (R1 to R5; table S5). OH radicals interacted with ozonolysis products and other species emitted from occupants and indoor surfaces (R12 to R16). Exponentially reduced emission rates of skincare products were applied to uncovered skin (table S2). When two occupants applied fragrances to their hands, monoterpenes, linalool, ethanol, and acetaldehyde were released. Under lotion conditions, with four occupants wearing lotion on uncovered skin surfaces, ethanol and phenoxyethanol were emitted. These emitted species then interacted with ozone and OH radicals (R10 and R11 and R21 to R24).

The CFD model used the Menter k-ω shear stress transport model to simulate turbulent airflow generated by thermal plumes around occupants (*11*). Model validation was conducted by comparing time-varying concentrations of various species, such as ozone and skincare products (acetaldehyde, ethanol, monoterpene, and phenoxyethanol), along with isoprene. Figure S15 illustrates these comparisons, with red dots representing model results and black dots indicating experimental values. The concentrations of ozone and species in the CFD model closely mirrored the experimental trends.

## Supplementary Materials

### The PDF file includes:

Supplementary Text Figs. S1 to S15 Tables S1 to S6 Legends for movies S1 to S4 Legend for data S1
