# Supplementary material for: Personal care products disrupt the human oxidation field
Source: Sci Adv. 2025 May 21;11(21):eads7908. doi: 10.1126/sciadv.ads7908 (PMC12094237; doi:10.1126/sciadv.ads7908)
Supplement: Supplementary file 1 — Supplementary Text Figs. S1 to S15 Tables S1 to S6 Legends for movies S1 to S4 Legend for data S1 [file sciadv.ads7908_sm.pdf]

Science Advances
AAAS

Supplementary Materials for

Personal care products disrupt the human oxidation field

Nora Zannoni *et al.*

Corresponding author: Jonathan Williams, jonathan.williams@mpic.de; Nora Zannoni, nora.zannoni@mpic.de; Manabu Shiraiwa, m.shiraiwa@uci.edu; Donghyun Rim, dxr51@psu.edu

Sci. Adv. **11**, eads7908 (2025)
DOI: 10.1126/sciadv.ads7908

The PDF file includes:

Supplementary Text
Figs. S1 to S15
Tables S1 to S6
Legends for movies S1 to S4
Legend for data S1

Other Supplementary Material for this manuscript includes the following:

Movies S1 to S4
Data S1

Supplementary Text

Results

Indoor air chemistry of people wearing a body lotion or a fragrance

The conceptual framewok of this study is showed in fig.S1.

The speciated contribution to the measured OH reactivity during the O_3-free condition (fig. S2A) of four adult volunteers wearing a lotion is dominated by isoprene (emitted from the occupants breath) and phenoxyethanol (emitted from the lotion). In contrast, 6-MHO (emitted from skin) dominates the OH reactivity under O_3-condition (fig. S2B), followed by phenoxyethanol (from the lotion), 6-MHO-OH (from skin), isoprene (from breath) and 4-OPA (from skin). This remarks the primary role of 6-MHO as a reactive molecule indoors, even when its production is inhibited due to the effective dilution of squalene with the body lotion components.

The model predicts that the application of lotions would cause a large decrease in OH concentrations over the time investigated (Fig. 2). At longer times the decrease is relatively consistent with calculated steady-state values of ~34 % (table S1). OH reactivity and concentrations at shorter times will be influenced more by the rapid emission of phenoxyethanol from the lotion which reaches its maximum concentration at an earlier time than 6-MHO (Fig. 2). This can cause OH concentrations to decrease by up to 140%.

A widely used perfume was chosen as a representative fragrance worn by people to study how the chemical composition of a fragrance can influence the human OH field indoors. The chemical composition of the fragrance from the moment of being sprayed inside the chamber by the volunteers and speciated into the terpenoids and the total monoterpenes is reported in fig. S3 and fig. S4, respectively. Figure S3 shows the evolution of the terpenoid concentration composition between the first 10 minutes when the fragrance was applied inside the room (O_3 had not been introduced), and 30 minutes later (after O_3 was introduced). The off-line analysis was combined with the real-time measurements conducted with proton transfer reaction mass spectrometry (PTR-MS) that is not separating the isomer compounds, therefore measures the different monoterpenes ($C_{10}H_{16}$) at the same *m/z* 137.133025, as a sum of the total monoterpenes. To simplify the calculations, we considered the weighted contributions of the monoterpenes having a concentration above 100 ppt at the moment the fragrance was applied, resulting in the following: limonene (77%), β-pinene (10%), γ-terpinene (6%), α-pinene (6%), β-myrcene (1%). This composition yielded a reaction rate constant between the summed monoterpenes concentration measured by PTR-MS and ozone equal to 1.91×10^{-16} cm^3 $molecule^{-1}$ s^{-1} and an OH yield of 0.81. The ozone reactivities and OH production rates speciations represented in fig. S3B-C reflect the combined effect of abundances and reaction rates of the considered monoterpenes (note for example the larger contribution in reactivity and yield of the less abundant β-myrcene compared with the more abundant β-pinene).

Limonene, β-pinene, γ-terpinene and o-cymol were the most abundant terpenoids found in the sample taken while the fragrance was applied. Interestingly, the optical enantiomer (+) limonene, known for having a remarkable citrous smell, was the dominant molecule half an hour after the fragrance was applied. We have not investigated this aspect further, but is important to notice that optical enantiomers have the same gas-phase reactivity towards achiral chemical molecules, such

as O_3 (see also the experiments conducted in Zannoni et al. (*51*)). Figure S4 shows the speciated monoterpene fraction found in the sample taken at 335 min (time when the fragrance was applied), which was used to determine a weighted reaction rate coefficient of the total monoterpenes with O_3 and the OH production rate used for inferring the OH concentration from the monoterpenes' contribution. Limonene, β-pinene and γ-terpinene were the most concentrated monoterpenes present in the fragrance, while ~50 % of the total O_3 reactivity and OH production rate can be explained solely by limonene (fig. S4).

Gas-phase partitioning of the measured species

Multiple factors influence the indoor OH production and OH loss (OH reactivity) during the use of personal care products. These include the chemical composition of the fragrance or lotion, the reaction rate coefficients with O_3 and OH radicals, and the efficiency with which each compound generates OH when reacting with ozone. Indoor-to-outdoor mass transport is also important in controlling the concentrations of species indoors. The e-folding time of the emissions, which can be calculated using the equations shown in table S2, are also important. For example, phenoxyethanol which is emitted from lotions has a long emission e-folding time and is emitted slowly over time. In contrast, the more volatile ethanol has a much shorter emission e-folding time as it partitions to the gas phase quickly leading to rapid removal by indoor-to-outdoor mass transport, while on the time scale of the experiment the decrease appears to be quasi-linear. Figure S9 shows the e-folding time determined by the model fitting which has been plotted against the vapor pressures of the different compounds. A clear trend is observed for the species emitted by the fragrances. Species with a lower vapor pressure are emitted at a slower rate and have a longer emission e-folding time. The emission e-folding times for species emitted from the lotions are significantly higher than for the fragrances, indicating that emissions may be slowed down by diffusion limitations or interactions occurring in the lotions. This is what we would expect as consumers, to wear a lotion that remains on the skin, and to wear a fragrance that evaporates for being perceived. In order to capture the full extent of the perfume application, from the initial highly volatile top notes through to the more persistent middle and base notes we opted for applying the fragrance on the volunteers just before they entered the chamber. Figure S12 shows the rate of decrease of the measured *m/z* inside the chamber while volunteers were sitting inside, wearing a perfume applied before entering the chamber, without ozone being injected. Different rates were observed, depending on the measured masses, indicating that compounds had different residence times.

Figures

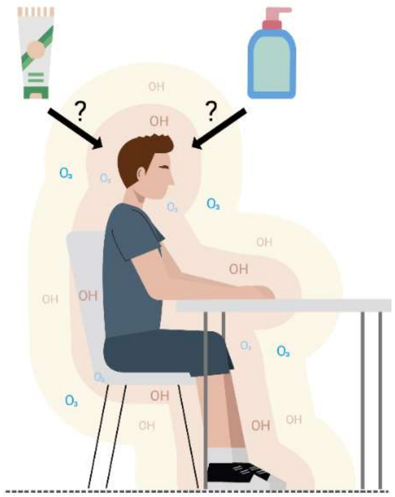

Figure S1. Conceptual framework of the experiment. In the benchmark condition one young adult sitting in a room generates an OH field from the ozonolysis reactions occurring on his skin surface when ozone infiltrates from outdoors. The experiment aims at investigating how the natural OH field is impacted when this person uses a personal care product such as a body lotion or a perfume in the room.

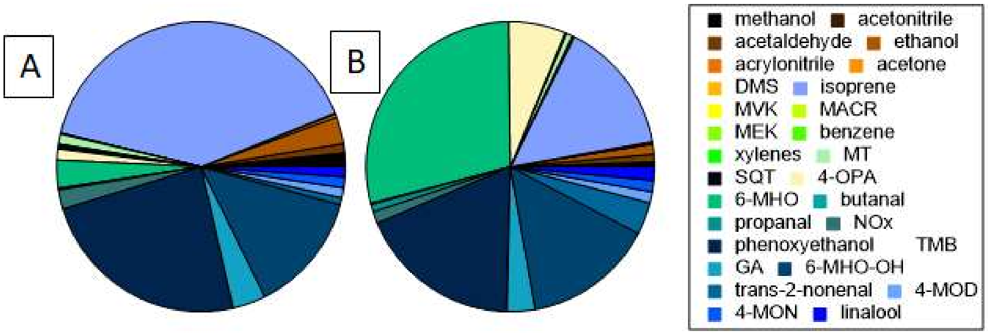

Figure S2. OH reactivity speciation fragrance study-case. A. Measured OH reactivity chemical speciation of four adult volunteers wearing a lotion during O_3-free conditions. B. Measured OH reactivity chemical speciation of four adult volunteers wearing a lotion during the condition with O_3. The speciation was calculated when concentrations were at steady state (15 minutes of the experiment before people left the room). DMS stands for dimethylsulfide, MVK, MACR and MEK stand for methylvinylketone, methacrolein, and methylethylketone. MT and SQT stand for total monoterpenes and total sesquiterpenes, respectively. 4-OPA, 6-MHO, GA, 6-MHO-OH, 4-MOD and 4-MON stand for 4-oxopentanal, 6-methyl-5-hepten-2-one, geranyl acetone, OH-6-methyl-5-hepten-2-one, 4-methyl-4-octene-1,8-dial and 4-methyl-8-oxo-4-nonenal.

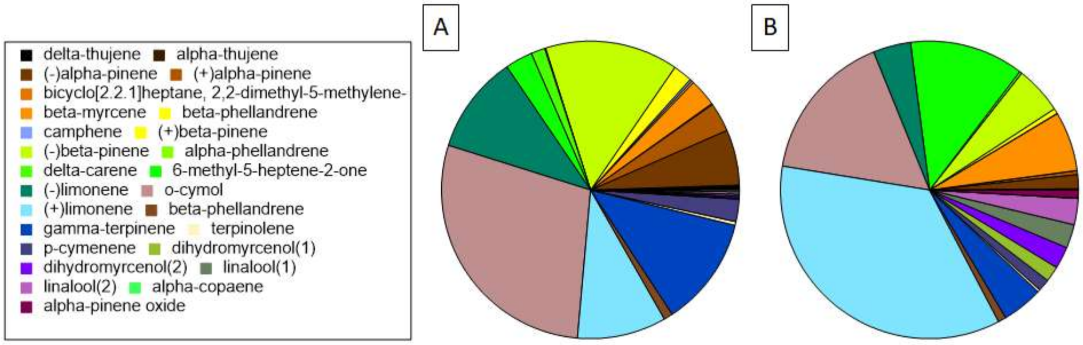

Figure S3. Terpenoid concentration during the fragrance study-case. Terpenoid chemical speciation of four volunteers wearing a fragrance. A. Terpenoid composition when the fragrance was sprayed inside the chamber (sample taken for 10 minutes from minute 332 to 342 in Fig.4) and B. Terpenoid composition half an hour after the fragrance was sprayed (sample taken for 10 minutes from min 368 to min 378 in Fig.4).

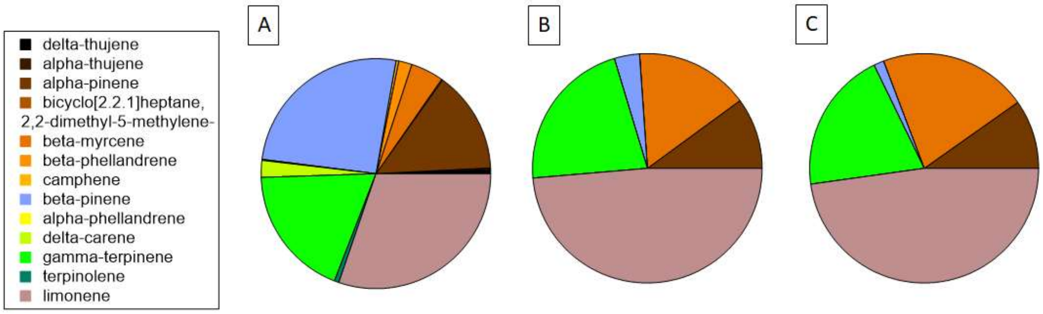

Figure S4. Monoterpenes reactivities and OH production rates in the fragrance study-case. Speciated monoterpene fraction for: (A) concentration determined in (ppt), (B) ozone reactivity in (s^{-1}), (C) OH production rate in (molecules cm^{-3} s^{-1}) at the time the fragrance was sprayed inside the chamber (values integrated for 10 minutes sampling starting at minute 332, see Fig.4). To simplify the calculations of B and C we assumed negligible the concentrations below 100 ppt reported in A.

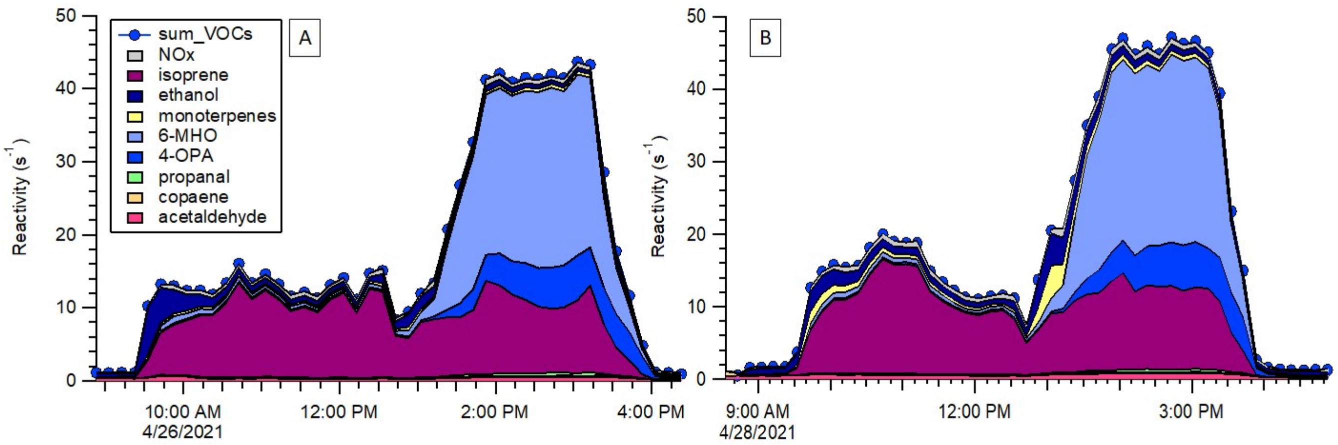

Figure S5. OH reactivity chemical speciation during the fragrance study-case. OH reactivity chemical speciation of four adult volunteers occupying the chamber under O_3-free and O_3 conditions. A) Represents the benchmark condition where no fragrance was worn by the volunteers. B) Represents the condition where a fragrance was applied on the back of the hands of two volunteers before entering the chamber, in the morning, and repeated in the afternoon followed by the introduction of O_3. The same group of volunteers participated in the two experiments. In both experiments, volunteers entered the chamber in the morning at 9:32, left for the lunch break at 12:30, entered in the afternoon at 12:49 (A) and 12:51 (B), and O_3 was introduced into the chamber at 13:00. A weighted reaction rate constant based on the fragrance composition was considered for the total monoterpene contribution to the OH reactivity, while the measured sesquiterpene was essentially one molecule, namely α-copaene.

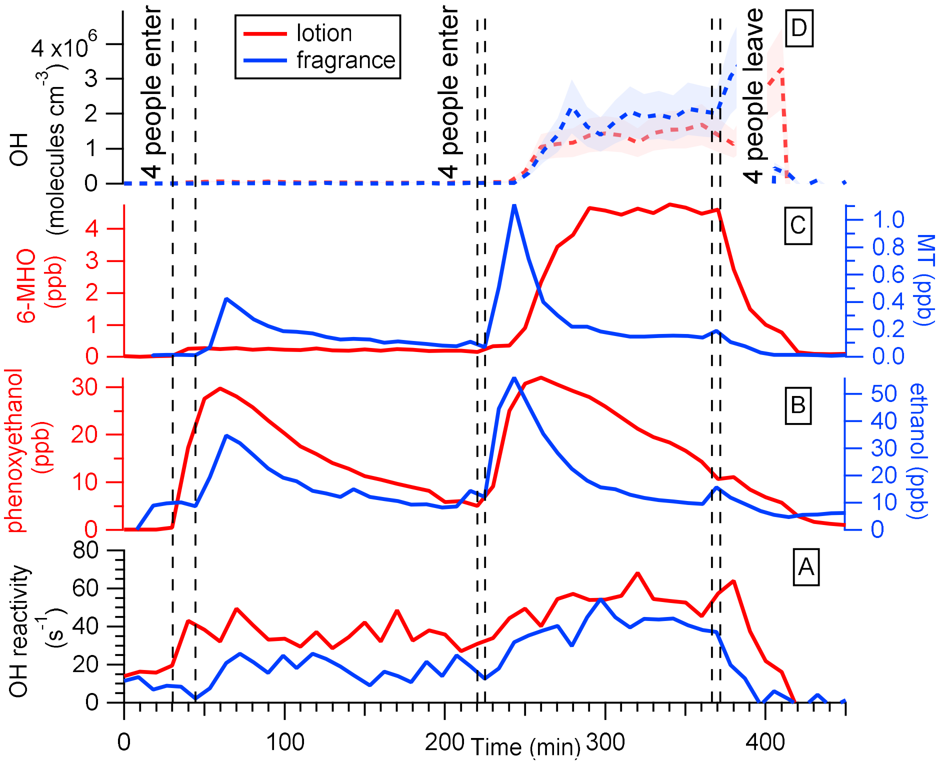

Figure S6. Comparison of the experimental results from the lotion study-case and the fragrance study-case. Four young adult volunteers wearing a body lotion occupy the chamber (red lines). Ozone (40 ppb) is mixed with chamber air starting at 240 min. A. OH reactivity, B. phenoxyethanol concentration, C. 6-MHO concentration, D. OH and ozone concentrations. The OH concentration was estimated with the steady-state method. In a different experiment, four young adult volunteers wearing a complex fragrance, including ethanol as a carrier, occupy the chamber (blue lines). The fragrance was applied on the back of the hands of two volunteers before entering the chamber in the morning (O_3-free condition) and before entering the chamber in the afternoon (O_3 condition). A. OH reactivity, B. Ethanol concentration, C. Sum of monoterpene concentrations, D. OH concentration. The estimated uncertainties on the measurements are: 48% (OH reactivity), 10-50% (phenoxyethanol, sum of monoterpenes, ethanol, 6-MHO), 35% (calculated OH concentration).

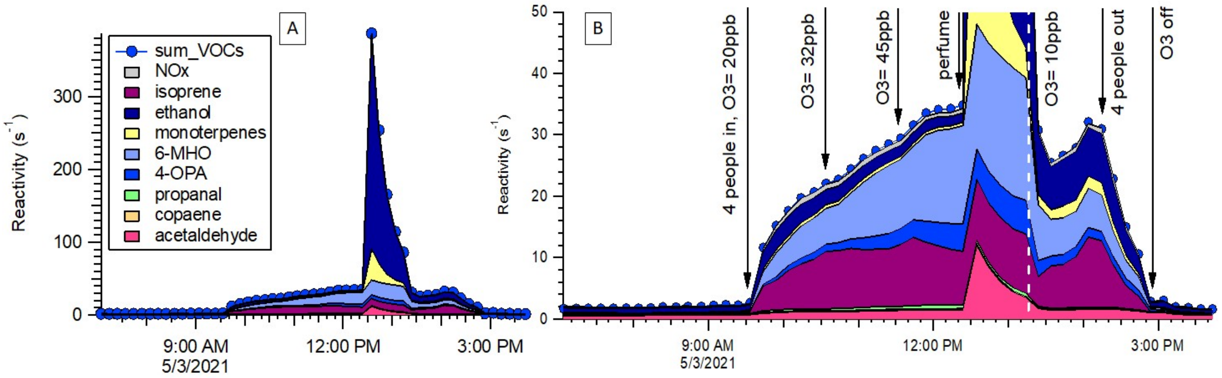

Figure S7. OH reactivity chemical speciation during the fragrance study-case. OH reactivity chemical speciation of four adult volunteers occupying the chamber under step-wise increasing concentrations of O_3 and O_3-free conditions, wearing a fragrance while in the room. The panels A and B show the same experiment, with panel B showing a zoomed-in screenshot of A with the experiment timeline. For comparison, the same group of volunteers was investigated in the experiments reported in fig. S4 and fig. S5. A weighted reaction rate constant based on the fragrance composition was considered for the total monoterpenes contribution to the OH reactivity, while the measured sesquiterpene was essentially α-copaene.

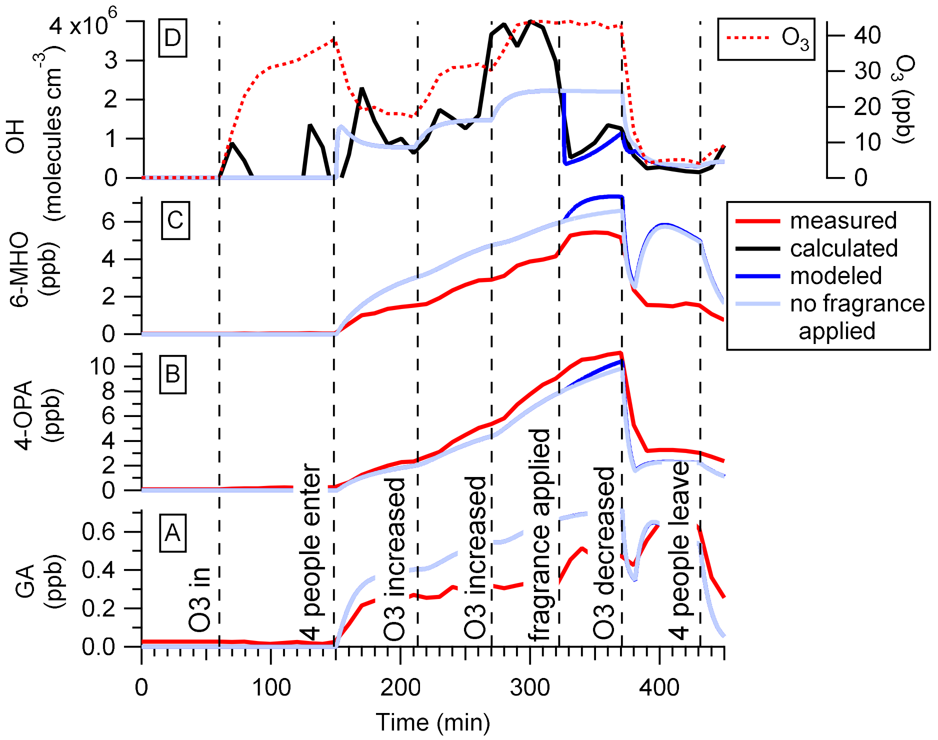

Figure S8. Volatile organic compounds concentrations measured during the fragrance study-case. Four adult volunteers wearing a fragrance with changing concentration of ozone. Concentrations of A. Geranyl acetone, B. 4-OPA, C. 6-MHO, and D. OH and ozone.

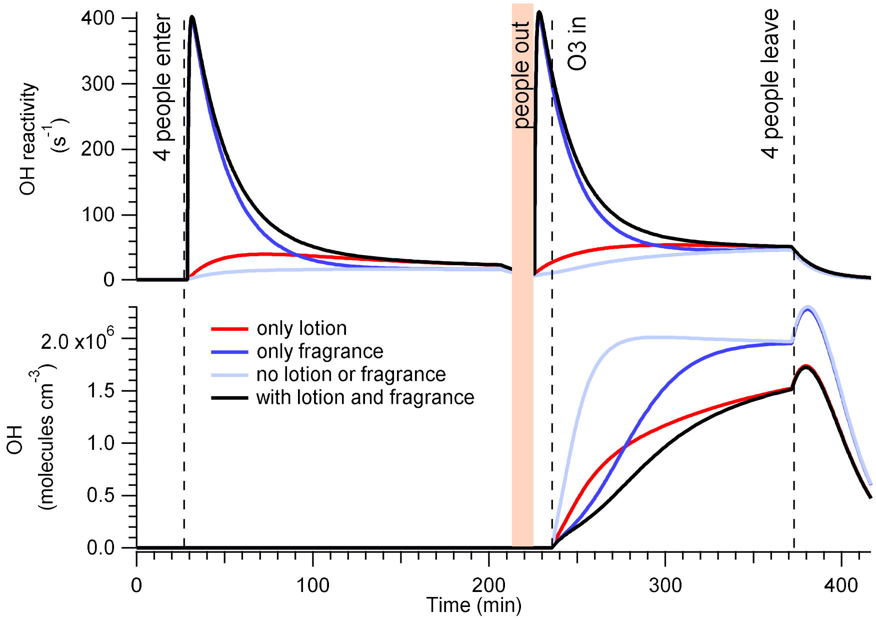

Figure S9: Modeled OH reactivity and concentration from the combined effect of wearing a lotion and a fragrance. The effect of wearing a lotion and fragrance on the OH reactivity and the OH concentration as simulated using KM-SUB-Skin-Clothing. The simulations show results with people simultaneously wearing the lotion and fragrance (black line), wearing no lotions or fragrances (cyan line), wearing only the lotion (red line) and wearing only a fragrance (blue line). For simulations with the fragrance, it was assumed that this was sprayed immediately upon people entering the chamber.

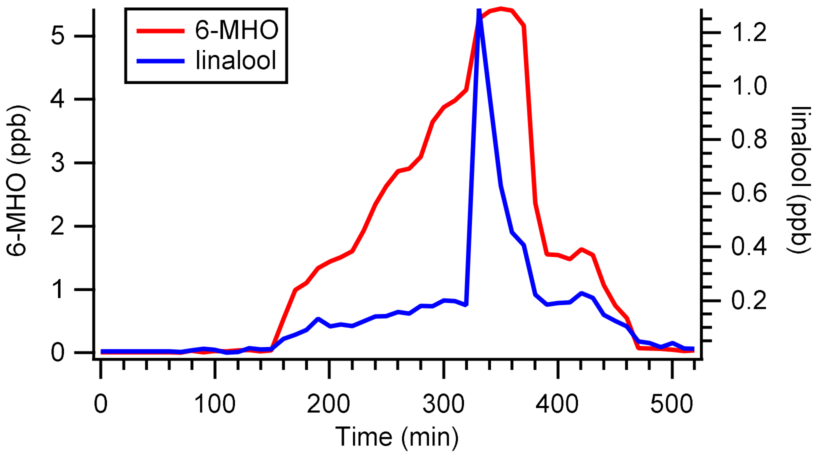

Figure S10. Measured 6-MHO and linalool concentrations emitted from four volunteers wearing a fragrance. 6-MHO is mainly emitted from ozonolysis reactions occurring on volunteers skin, with a negligible contribution from the ozonolysis reaction of linalool contained in the fragrance.

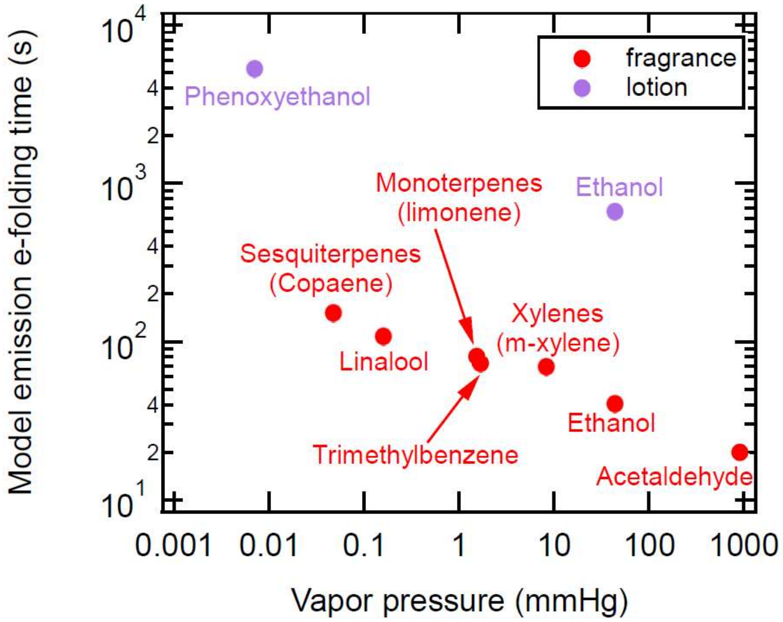

Figure S11. The model emission e-folding time as a function of vapor pressure. The emission e-folding time for each compound is calculated from the equations shown in table S1.

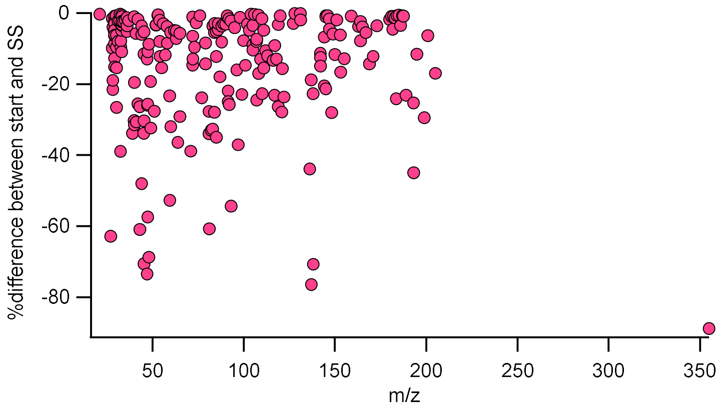

Figure S12. **Time-lasting effect of different volatile organic compounds composing the fragrance.** Rate of decrease (in %) of all the masses measured with PTR-ToF-MS during one experiment when volunteers applied a fragrance before entering the chamber in the absence of ozone inside the chamber. Data were filtered to represent only the masses that showed a decreased concentration from the start of the experiment (first 10 minutes after the volunteers entered the chamber) to the end of the experiment (last 10 minutes before volunteers left the chamber).

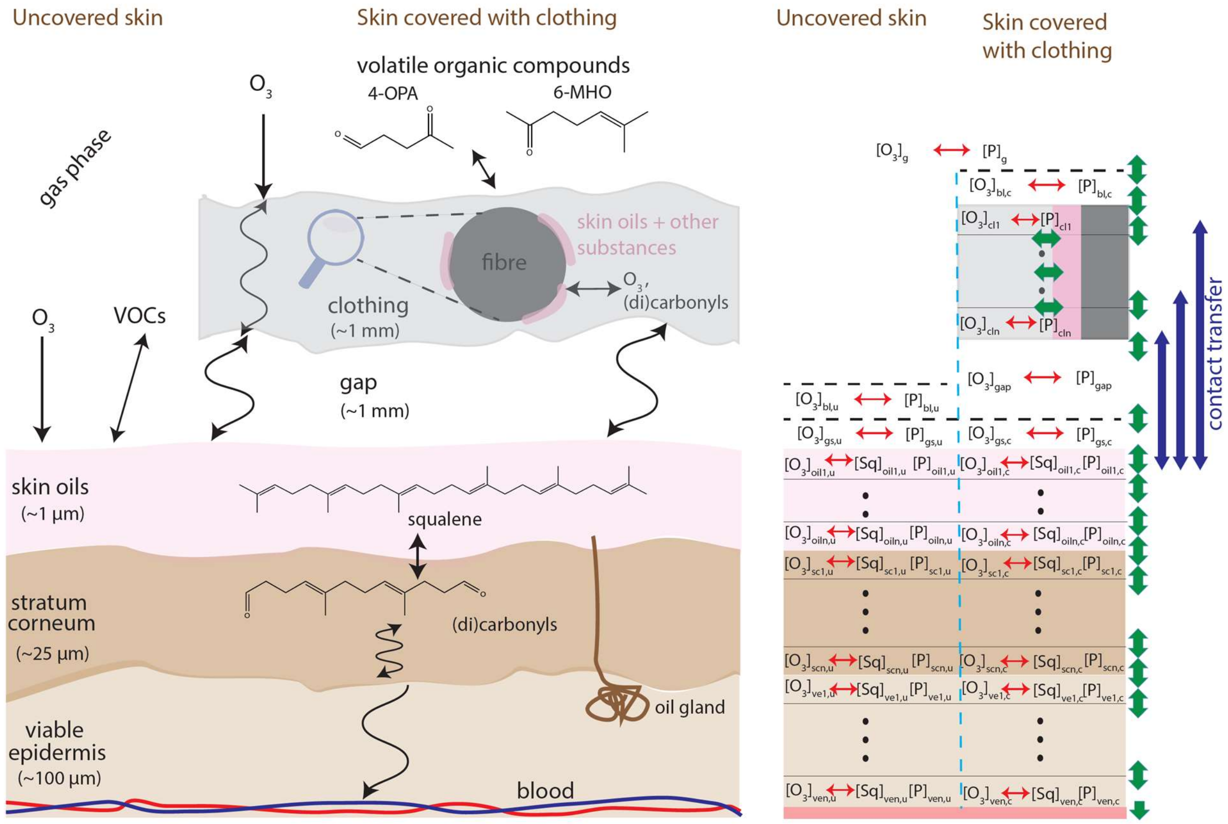

Figure S13. Kinetic model schematic. A schematic of the KM-SUB-Skin-Clothing model which has been adapted to include both uncovered skin and skin covered with clothing.

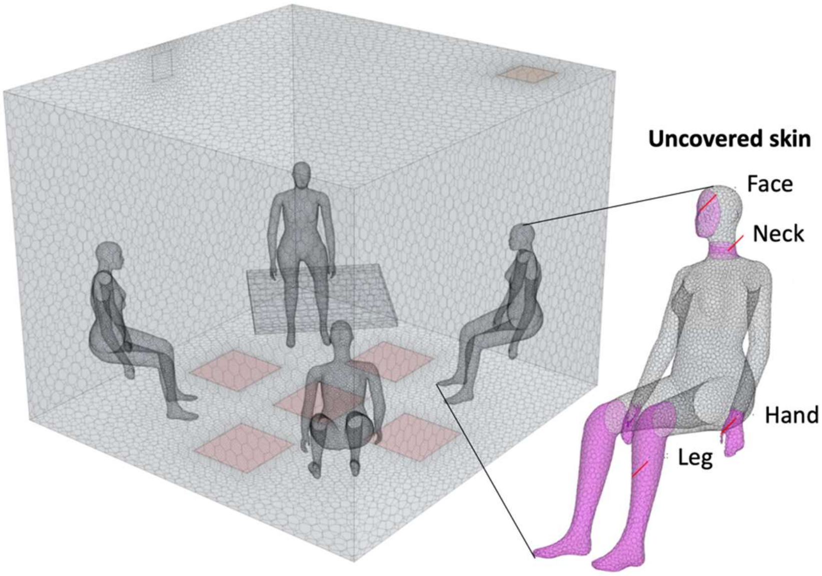

Figure S14. Computational fluid dynamics model geometry.

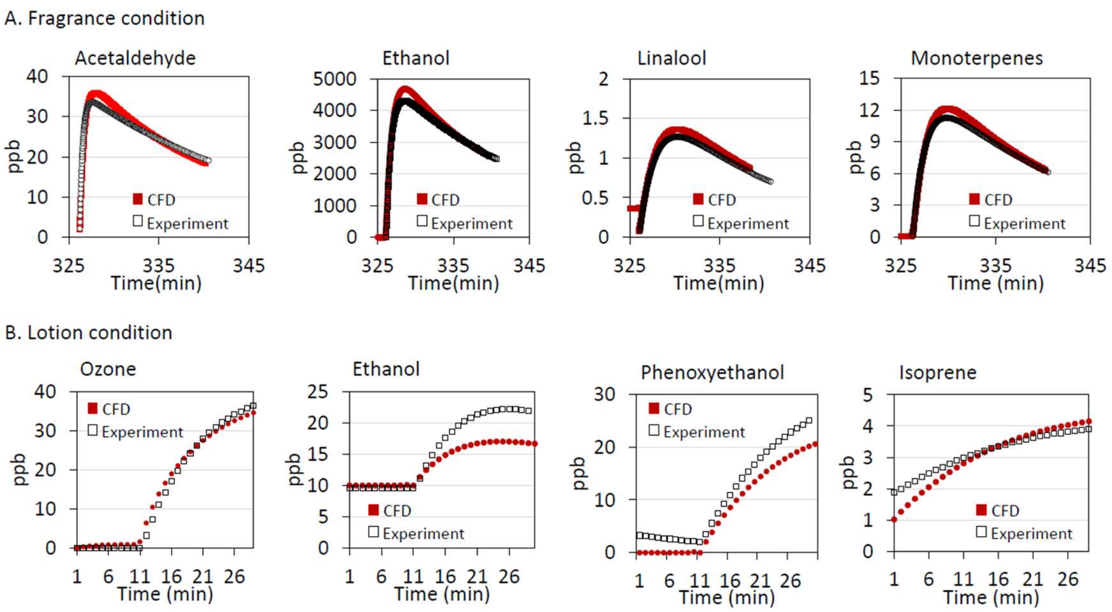

Figure S15. Computational fluid dynamics model validation with measured values. Comparison between the mixing ratios obtained from measurements with those obtained from the CFD model for some compounds, when the fragrance was applied (A) and when the lotion was applied (B).

Table S1. Summary of experimental results. Measured steady-state mean values inside the occupied chamber during the experiments involving two different groups of 4 adult volunteers (group 1 and group 2), the application of a body lotion and the application of a fragrance, all O_3-present conditions. Two replicate experiments were conducted with the same group of volunteers (group 2) applying the fragrance before entering the chamber. Max O_3 level refers to the condition of the maximum O_3 level reached inside the chamber during the experiment of volunteers applying the fragrance while sitting inside the chamber, before they application. OH concentration was obtained from measured alkene compounds and O_3 concentrations, and OH reactivity. MT refers to the sum of monoterpenes, GA refers to geranyl acetone.

**Experimental Condition**	O_3 [ppb]	OH reactivity [s^{-1}]	OH [molecules/cm^3]	MT [ppb]	Isoprene [ppb]	6-MHO [ppb]	GA [ppb]	4-MOD [ppb]	4-MON [ppb]	Linalool [ppb]
Benchmark GROUP 1	43.25	46	2.34×10^6	0.166	3.557	6.937	1.012	0.364	0.329	0.304
lotion applied	45.17	52	1.55×10^6	0.105	3.710	4.577	0.499	0.191	0.196	0.222
Benchmark GROUP 2	38.67	34	2.81×10^6	0.117	4.162	6.197	0.520	0.133	0.087	0.259
fragrance applied before entering chamber_1	40.83	41	2.02×10^6	0.149	4.501	6.521	0.583	0.156	0.112	0.307
fragrance applied before entering chamber_2	41.20	45	2.21×10^6	0.146	4.872	6.971	0.634	0.160	0.122	0.307
max O_3 level	43.60	17	3.73×10^6	0.127	3.917	3.837	0.332	0.103	0.054	0.193
fragrance applied while in the chamber	42.95	80	1.16×10^6	2.067	3.925	5.333	0.475	0.154	0.179	0.497

Table S2. Emission rates from fragrances and lotions used in the KM-SUB-Skin-Clothing model.

Compound	Emission rate to the gas phase (cm^{-3} s^{-1})	Notes	Included in CFD simulations?
**Fragrance emissions:**			
Ethanol	$2.92 \times 10^{12} \times \exp(-0.0246 \times t)$	For the experiment on the 3rd of May *t* is the time from which the fragrance is sprayed in the chamber. For the experiment on the 28th April *t* is the time from which the fragrance is sprayed. This is assumed to be 220 seconds and 180 seconds before the people enter the chamber for the first and second times that the fragrance is sprayed, respectively. These times are fitting parameters which were varied until the best fit to the measurements were obtained.	Yes
Trimethylbenzene	$3.48 \times 10^{7} \times \exp(-0.0136 \times t)$		No
Sesquiterpenes	$1.85 \times 10^{7} \times \exp(-6.54 \times 10^{-3} \times t)$		No
Xylenes	$2.77 \times 10^{7} \times \exp(-0.0143 \times t)$		No
Acetaldehyde	$4.15 \times 10^{10} \times \exp(-0.05 \times t)$		Yes
Linalool	$3.70 \times 10^{8} \times \exp(-9.20 \times 10^{-3} \times t)$		Yes
Monoterpenes	$4.28 \times 10^{9} \times \exp(-0.0123 \times t)$		Yes
**Lotion emissions:**			
Phenoxyethanol	$9.41 \times 10^{8} \times \exp(-1.883 \times 10^{-4} \times t)$	*t* is the time from which people enter the chamber.	Yes
Ethanol	$9.68 \times 10^{8} \times \exp(-1.5 \times 10^{-3} \times t)$		Yes

Table S3. Background breath and skin emission rates used in the KM-SUB-Skin-Clothing model.

Compound	Emission rate to the gas phase (cm^{-3} s^{-1})	Notes	Included in CFD simulations?
**Background emissions (always present):**			
Acetaldehyde	1.5×10^7 (Lotions experiment)<br><br>3.0×10^7 (Fragrance experiments)	Background concentrations were different for the different experiments.	Yes
Acetonitrile	$6.1 \times 10^5 \times$ AER (Lotions experiment)<br><br>$1.2 \times 10^6 \times$ AER (Fragrance experiments)	Background signal is likely to be coming from outdoor-to-indoor transport as the signal doesn't change when the air-exchange rate changes for the experiment on the 3rd May.<br><br>Background concentrations were different for different experiments.<br><br>Air exchange rate (AER) is in unit of h^{-1} in this equation and usually has a value of 2.93 h^{-1}	No
Ethanol	1.9×10^8		Yes
Acrylonitrile	1.0×10^5		No
Butanal	4.0×10^5		No
Methanol	1.0×10^8		No
NO	3.8×10^6		Yes
**Breath emissions (occurs in the presence of people):**			
Isoprene	9.6×10^7		Yes
Methanol	2.5×10^8 (Lotions experiment and 28th April fragrance experiment)<br><br>3.8×10^8 (3rd May fragrance experiment)	Steady state values varied between experiments.	No
Acetone	4.2×10^8 (Lotions experiment)<br><br>2.1×10^8 (Fragrance experiments)	Steady state values in the absence of ozone varied between experiments.	No
NO	2.5×10^7		Yes
NO_2	6.3×10^6		No
Trimethylbenzene	4.8×10^5		
Dimethylsulfide	5.0×10^6 (Lotions experiment and 28th April fragrance experiment)<br><br>7.6×10^6 (3rd May fragrance experiment)	Steady state values varied between experiments.	No
**Skin emissions (occurs in the presence of people):**			
Benzene	1.6×10^6 (Lotions experiment and 3rd May fragrance experiment)<br><br>8.0×10^5 (28th April fragrance experiment)	Steady state values varied between experiments.	No
Xylenes	9.6×10^5		No
Monoterpenes	3.2×10^6		Yes

Sesquiterpenes	1.9×10^6		No
MEK	$7.6 \times 10^5 + 1.5 \times 10^{-6} \times [O_3]_g$	A clear increase is observed in the concentrations of these species in the presence of ozone. This is a simplified way of treating this dependence.	No
6-MHO-OH	$8.0 \times 10^6 + 5.8 \times 10^{-5} \times [O_3]_g$		Yes
Linalool	2.4×10^6		Yes
MVK	9.6×10^5		No
Methacrolein	3.8×10^5		No

Table S4. Parameters used in the KM-SUB-Skin-Clothing model. Note that if parameters are not listed below, they will be identical to our previous publications (*5*, *19*).

Parameter	Value	Comment
Air exchange rate (h^{-1})	2.93	From measurements
Surface area of the 4 people (m^2)	7.72 (Lotions experiment)<br><br>8 (Fragrances experiments)	From measurements
Fraction of skin not covered by clothing	0.23 (Lotions experiment)<br><br>0.19 (Fragrances experiments)	From measurements
Ozone production rate (molecule cm^{-3} s^{-1})	Calculated as Inlet air concentration (ppb) × AER.	Maintains a concentration equivalent to the inlet air concentration in the chamber in the absence of people and wall losses.
Boundary layer next to the clothing (cm)	0.4 (Lotions experiment and 3rd May fragrance experiment)<br><br>0.5 (28th April fragrance experiment)	Fitting parameters. Relatively small boundary layers were consistent with turbulent air.
Boundary layer next to the uncovered skin (cm)	0.65	Fitting parameter. A relatively small boundary layer was consistent with turbulent air.
Initial concentration of squalene in skin oil mixed with the lotion (cm^{-3})	1.05×10^{19}	See text
Initial concentration of other reactive species in skin oil mixed with the lotion (cm^{-3})	1.05×10^{19}	See text
Effective squalene conc. in the clothing skin oil and other substances (cm^{-3})	5×10^{18}	Concentrations were fitting parameters and are within a factor of 5 of (*5*)
Effective conc. of other skin oil reactive species in the clothing skin oil and other substances (cm^{-3})	2.2×10^{18}	
Effective conc. of laundering species 1 in the clothing skin oil and other substances (cm^{-3})	5.6×10^{17}	
Effective conc. of laundering species 2 in the clothing skin oil and other substances (cm^{-3})	2.8×10^{18}	
Yield of propanal from the reaction of other skin oil reactive species with ozone	0.11	Fitting parameters.
Yield of trans-2-nonenal from the reaction of other skin oil reactive species with ozone	0.16	
Yield of acetaldehyde from the reaction of other skin oil reactive species with ozone	0.07	
Yield of butanal from the reaction of other skin oil reactive species with ozone	0.02	
Yield of trans-2-nonenal from the first order loss of ozone to lotions	0.15	
First order loss rate coefficient of ozone reacting with lotions (cm^3 s^{-1})	1.88×10^4	Fitting parameter. Also see notes on a previous slide. (For comparison, this is similar to the first order rate coefficient for squalene reacting with ozone in skin oil in the absence of lotions)
Partitioning coefficient of butanal for clothing (mol cm^{-3} atm^{-1})	0.0014	Assumed to be the same as the value used for acetone, acetaldehyde and propanal.
Partitioning coefficient of trans-2-nonenal for clothing (mol cm^{-3} atm^{-1})	6.4	These were changed in order to better fit the data. Changes were no more than

Partitioning coefficient of geranyl acetone for clothing (mol cm^{-3} atm^{-1})	1.5×10^{3}	a factor of 2.5 compared to previous values.
Partitioning coefficient of 4-MON for clothing (mol cm^{-3} atm^{-1})	1.1×10^{3}	
Initial concentration of geranyl acetone in the clothing skin oil and other substances (cm^{-3})	1.4×10^{18} (Lotions experiment)<br>7.8×10^{17} (Fragrances experiments)	These species are semi-volatile and may therefore be present from reactions occurring before the people enter the chamber. All other species are assumed to have an initial concentration of zero. It was necessary to include these to account for the increase in their gas-phase concentrations in the presence of people and absence of ozone.
Initial concentration of 4-MON in the clothing skin oil and other substances (cm^{-3})	2.5×10^{17} (Lotions experiment)<br>4.3×10^{16} (Fragrances experiments)	
Initial concentration of 4-MOD in the clothing skin oil and other substances (cm^{-3})	8.3×10^{16} (Lotions experiment)<br>2.7×10^{16} (Fragrances experiments)	
Initial concentration of trans-2-nonenal in the clothing skin oil and other substances (cm^{-3})	3.8×10^{15} (All experiments)	
Geranyl acetone production rate in clothing skin oil and other substances without ozone reactions (molecule cm^{-3} s^{-1})	2.5×10^{13} (Lotions experiment)<br>2.0×10^{13} (Fragrances experiments)	Needed to explain the increase and then steady concentration in the presence of people and with no ozone. May be due to previous reactions occurring before the people enter the chamber and the molecules being only semi-volatile. Possibly a constant production is due to transport from the skin oil or due to reactions of long-lived intermediates.
4-MON production rate in clothing skin oil and other substances without ozone reactions (molecule cm^{-3} s^{-1})	1.5×10^{12} (Lotions experiment)<br>3.9×10^{11} (Fragrances experiments)	
4-MOD production rate in clothing skin oil and other substances without ozone reactions (molecule cm^{-3} s^{-1})	5.8×10^{11} (Lotions experiment)<br>2.6×10^{11} (Fragrances experiments)	
Trans-2-nonenal production rate in clothing skin oil and other substances without ozone reactions (molecule cm^{-3} s^{-1})	4.6×10^{11} (All experiments)	
Propanal production rate in clothing skin oil and other substances without ozone reactions (molecule cm^{-3} s^{-1})	9.1×10^{11} (All experiments)	
4-OPA production rate in clothing skin oil and other substances without ozone reactions (molecule cm^{-3} s^{-1})	4.5×10^{12} (All experiments)	

Table S5. Gas-phase reactions included in the KM-SUB-Skin-Clothing model.

	Reaction	Rate coefficient ($cm^3\ s^{-1}$)	OH Yield	Notes	Included in CFD simulations?
	**Reactions involving ozone:**				
1	6-MHO +O_3 → 4-OPA + Acetone + Y_1 OH	$k_1 = 4.3 \times 10^{-16}$	$Y_1 = 0.75$		Yes
2	Geranyl acetone + O_3 →0.5 4-OPA + 0.5 4-MON + 0.5 Acetone + 0.5 6-MHO + Y_2 OH	$k_2 = 8.6 \times 10^{-16}$	$Y_2 = 1$		Yes
3	4-MON + O_3 → 2 4-OPA + Y_3 OH	$k_3 = 4.3 \times 10^{-16}$	$Y_3 = 0.92$		Yes
4	4-MOD + O_3 → 4-OPA + 1,4 butanedial + Y_4 OH	$k_4 = 4.3 \times 10^{-16}$	$Y_4 = 0.92$		Yes
5	Trans-2-nonenal + O_3 → Product + Y_6 OH	$k_6 = 1.3 \times 10^{-17}$	$Y_6 = 1$		Yes
6	6-MHO-OH + O_3 → Product + Y_{11} OH	$k_{11} = 3.8 \times 10^{-16}$	$Y_{11} = 1$		Yes
7	Isoprene + O_3 → Product + Y_5 OH	$k_5 = 1.28 \times 10^{-17}$	$Y_5 = 0.27$		Yes
8	NO + O_3 → NO_2 (+ O_2)	$k_{14} = 1.9 \times 10^{-14}$	N/A	IUPAC recommended value	Yes
9	NO_2 + O_3 → Products	$k_{15} = 3.5 \times 10^{-17}$	N/A	IUPAC recommended value	Yes
10	Monoterpenes + O_3 → Product + Y_{10} OH	$k_{10} = 2.2 \times 10^{-16}$ (Lotions experiment)<br><br>$k_{10} = 1.91 \times 10^{-16}$ (Fragrance experiments)	$Y_{10} = 0.86$ (Lotions experiment)<br><br>$Y_{10} = 0.8083$ (Fragrance experiments)		Only fragrance
11	Linalool + O_3 → Product + 0.1 6-MHO + Y_{12} OH	$k_{12} = 4.1 \times 10^{-16}$	$Y_{12} = 0.72$		Only fragrance
	Acrylonitrile + O_3 → Product + Y_7 OH	$k_7 = 1.3 \times 10^{-19}$	$Y_7 = 1$		No
	MVK + O_3 → Product + Y_8 OH	$k_8 = 3.2 \times 10^{-18}$	$Y_8 = 0.18$		No
	MACR + O_3 → Product + Y_9 OH	$k_9 = 3.2 \times 10^{-18}$	$Y_9 = 0.18$		No
	Sesquiterpenes + O3 → Product + Y_{13} OH	$k_{13} = 1.6 \times 10^{-16}$	$Y_{13} = 0.38$		No
	Linalool + O_3 → 0.35 Acetone + 0.32 Formaldehyde + 0.28 Hydroxyacetone + Other products + 0.72 OH	$k_{16} = 4.1 \times 10^{-16}$			No
	**Reactions involving the OH radical:**				
12	6-MHO + OH → Product	$k_{16} = 1.57 \times 10^{-10}$	N/A		Yes
13	4-OPA + OH → Product	$k_{18} = 2 \times 10^{-11}$	N/A		Yes
14	Geranyl acetone + OH → Product	$k_{19} = 1.57 \times 10^{-10}$	N/A		Yes
15	4-MON + OH → Product	$k_{20} = 1.57 \times 10^{-10}$	N/A		Yes
16	4-MOD + OH → Product	$k_{21} = 1.57 \times 10^{-10}$	N/A		Yes
17	Isoprene + OH → Y_{14} MVK + Y_{15} Methacrolein	$k_{22} = 1 \times 10^{-10}$ ($Y_{14} = 0.22$ and $Y_{15} = 0.11$)	N/A	The yields of MVK and Methacrolein are from (*23*)	Yes
18	Propanal + OH → Product	$k_{23} = 2 \times 10^{-11}$	N/A		Yes
19	Trans-2-nonenal + OH → Product	$k_{26} = 4.35 \times 10^{-11}$	N/A		Yes
20	NO + OH → Product	$k_{40} = 1 \times 10^{-11}$	N/A		Yes
21	Acetaldehyde + OH → Product	$k_{24} = 1.5 \times 10^{-11}$	N/A		Only fragrance
22	Ethanol + OH → Product	$k_{28} = 3.2 \times 10^{-12}$	N/A		Yes
23	Monoterpenes + OH → Product	$k_{37} = 1.64 \times 10^{-10}$	N/A		Yes
24	Phenoxyethanol + OH → Product	$k_{42} = 3.27 \times 10^{-11}$	N/A		Only lotion
	Methanol + OH → Product	$k_{25} = 9 \times 10^{-13}$	N/A		No
	Acetone + OH → Product	$k_{17} = 1.8 \times 10^{-13}$	N/A		No
	Acetonitrile + OH → Product	$k_{27} = 2.2 \times 10^{-14}$	N/A		No
	Acrylonitrile + OH → Product	$k_{29} = 5 \times 10^{-12}$	N/A		No
	Dimethylsulfide + OH → Product	$k_{30} = 4.8 \times 10^{-12}$	N/A		No

	MVK + OH → Product	$k_{31} = 2.01 \times 10^{-11}$	N/A		No
	Methacrolein + OH → Product	$k_{32} = 2.86 \times 10^{-11}$	N/A		No
	MEK + OH → Product	$k_{33} = 1.1 \times 10^{-12}$	N/A		No
	Benzene + OH → Product	$k_{34} = 1.2 \times 10^{-12}$	N/A		No
	Xylenes + OH → Product	$k_{35} = 1.7 \times 10^{-11}$	N/A		No
	Trimethylbenzene + OH → Product	$k_{36} = 4.06 \times 10^{-11}$	N/A		No
	Sesquiterpenes + OH → Product	$k_{38} = 9 \times 10^{-11}$	N/A		No
	Butanal + OH → Product	$k_{39} = 2.35 \times 10^{-11}$	N/A		No
	NO_2 + OH → Product	$k_{41} = 3 \times 10^{-11}$	N/A		No
	Linalool + OH → 0.34 Acetone + 0.14 Glycolaldehyde + 0.1 6-MHO + 0.05 Formic acid + 0.028 Formaldehyde + 0.021 CO + Other products	$k_{43} = 1.7 \times 10^{-10}$	N/A		No
	Formaldehyde + OH → Products	$k_{44} = 8.5 \times 10^{-12}$	N/A		No
	Hydroxyacetone + OH → Products	$k_{45} = 3 \times 10^{-12}$	N/A		No
	Glycoaldehyde + OH → Products	$k_{46} = 8 \times 10^{-12}$	N/A		No
	Formic acid + OH → Products	$k_{47} = 4.5 \times 10^{-13}$	N/A		No
	CO + OH → Products	$k_{48} = 2.8 \times 10^{-12}$	N/A		No
	**First-order loss rate from the gas phase due to deposition to non-human surfaces (s^{-1}):**				
25	O_3 (+ surfaces) → Product	$k_{43} = 1 \times 10^{-4}$ (Lotions experiment and Fragrance experiment on the 28th April)<br><br>$k_{43} = 2 \times 10^{-4}$ (Fragrance experiment on the 3rd May)	N/A	These were all fitting values that were necessary to reproduce the data. Ozone and large molecules are expected to deposit to surfaces.	Yes
26	Geranyl acetone (+ surfaces) → Product	$k_{44} = 1 \times 10^{-3}$	N/A		Yes
27	4-MON (+ surfaces) → Product	$k_{45} = 1 \times 10^{-3}$	N/A		Yes
28	4-MOD (+ surfaces) → Product	$k_{46} = 1 \times 10^{-3}$	N/A		Yes

*CFD model included 24 chemical reactions for the lotion case and 27 reactions for the fragrance case.

Table S6. Ozone uptake coefficients and product yields outputted from the KM-SUB-Skin-Clothing model for use as input parameters in the CFD simulations. Note that these are average values over the time where ozone has reached a constant concentration.

	**Lotion experiment**	**Fragrance experiment (28th April)**	**Fragrance experiment (3rd May)**	**Lotion experiment<br><br>Lotion removed in the model**	**Fragrance experiment (28th April) Fragrance removed in the model**	**Fragrance experiment (3rd May) Fragrance removed in the model**	**Included in CFD simulations**
**Uptake coefficients for clothing*:**							
Ozone	3.62×10^{-4}	3.52×10^{-4}	3.26×10^{-4}	3.62×10^{-4}	3.52×10^{-4}	3.26×10^{-4}	Yes
**Uptake coefficients for uncovered skin*:**							
Ozone	2.40×10^{-3}	2.95×10^{-3}	3.12×10^{-3}	2.99×10^{-3}	2.95×10^{-3}	3.12×10^{-3}	Yes
**Yields for clothing**:**							
Butanal	0.003	0.003	0.003	0.003	0.003	0.003	No
Acetaldehyde	0.011	0.012	0.010	0.011	0.012	0.011	Yes
Propanal	0.021	0.021	0.020	0.021	0.021	0.020	Yes
Trans-2-nonenal	0.023	0.025	0.025	0.025	0.025	0.025	Yes
Acetone	0.264	0.257	0.253	0.264	0.257	0.252	No
6-MHO	0.192	0.189	0.214	0.189	0.189	0.218	Yes
Geranyl acetone	0.024	0.015	0.013	0.019	0.015	0.013	Yes
4-OPA	0.101	0.079	0.118	0.101	0.079	0.118	Yes
4-MON	0.005	0.001	0.002	0.005	0.001	0.002	Yes
4-MOD	0.007	0.004	0.007	0.006	0.004	0.007	Yes
**Yields for uncovered skin**:**							
Butanal	0.001	0.006	0.006	0.006	0.006	0.006	No
Acetaldehyde	0.002	0.022	0.020	0.022	0.022	0.020	Yes
Propanal	0.003	0.034	0.031	0.034	0.034	0.031	Yes
Trans-2-nonenal	0.140	0.049	0.045	0.049	0.049	0.045	Yes
Acetone	0.023	0.224	0.225	0.225	0.224	0.225	No
6-MHO	0.023	0.223	0.224	0.225	0.223	0.224	Yes
Geranyl acetone	0.014	0.179	0.193	0.181	0.179	0.192	Yes
4-OPA	0.002	0.013	0.029	0.015	0.013	0.029	Yes
4-MON	0.001	0.006	0.014	0.007	0.006	0.014	Yes
4-MOD	0.002	0.013	0.028	0.015	0.013	0.028	Yes

* Calculated using the near surface gas-phase concentration

** Calculated as (Flux of VOC out of the clothing or skin - flux of VOC into the clothing or skin)/(Flux of ozone into the clothing or skin-flux of O_3 out of the clothing or skin) using the near surface gas phase concentration.

Movie S1. Ethanol dispersion from the uncovered skin of four occupants following the introduction of ozone into the room. Ozone was introduced through an inlet at the floor level. The ethanol dispersion is tracked at each second for 10 minutes, with each timestep representing 1 second.

Movie S2. Phenoxyethanol dispersion from the uncovered skin of four occupants following the introduction of ozone into the room. Ozone was introduced through an inlet at the floor level for 10 minutes. The Phenoxyethanol dispersion is tracked at each second for 10 minutes, with each timestep representing 1 second.

Movie S3. OH radical removal around the occupants when spraying the fragrance on the back of the hands of two occupants sitting in the chamber. The removal is tracked at each second for 3 minutes, with each timestep representing 1 second.

Movie S4. OH reactivity field around the occupants when spraying the fragrance on the back of the hands of two occupants sitting in the chamber. OH reactivity is tracked at each second for 3 minutes, with each timestep representing 1 second.

Data S1. (separate file)
Summary of data. Summary of data including gases concentrations, OH reactivity and OH radical concentration for the lotion experiment and the fragrance experiments. The no-fragrance and no-lotion simulations results are reported with the data.
